# Multimorbidity, polypharmacy, and drug-drug-gene interactions following a non-ST elevation acute coronary syndrome: analysis of a multicentre observational study

**DOI:** 10.1186/s12916-020-01827-z

**Published:** 2020-11-25

**Authors:** R. M. Turner, E. M. de Koning, V. Fontana, A. Thompson, M. Pirmohamed

**Affiliations:** 1 Wolfson Centre for Personalised Medicine, University of Liverpool, Liverpool, L69 3GL UK; 2 Franciscus Gasthuis & Vlietland, Rotterdam, the Netherlands

**Keywords:** Multimorbidity, Polypharmacy, Interactions, Pharmacogenomics, Acute coronary syndrome

## Abstract

**Background:**

The number of patients living with co-existing diseases is growing. This study aimed to assess the extent of multimorbidity, medication use, and drug- and gene-based interactions in patients following a non-ST elevation acute coronary syndrome (NSTE-ACS).

**Methods:**

In 1456 patients discharged from hospital for a NSTE-ACS, comorbidities and multimorbidity (≥ 2 chronic conditions) were assessed. Of these, 698 had complete drug use recorded at discharge, and 652 (the ‘interaction’ cohort) had drug use and actionable genotypes available for *CYP2B6*, *CYP2C9*, *CYP2C19*, *CYP2D6*, *CYP3A5*, *DPYD*, *F5*, *SLCO1B1*, *TPMT*, *UGT1A1*, and *VKORC1*. The following drug interactions were investigated: pharmacokinetic drug-drug (DDIs) involving CYPs (CYPs above, plus CYP1A2, CYP2C8, CYP3A4), SLCO1B1, and P-glycoprotein; drug-gene (DGIs); drug-drug-gene (DDGIs); and drug-gene-gene (DGGIs). Interactions predicted to be ‘substantial’ were defined as follows: DDIs due to strong inhibitors/inducers, DGIs due to variant homozygous/compound heterozygous genotypes, and DDGIs/DGGIs where the constituent DDI/DGI(s) both influenced the victim drug in the same direction.

**Results:**

In the whole cohort, 727 (49.9%) patients had multimorbidity. Non-linear relationships between age and increasing comorbidities and decreasing coronary intervention were observed. There were 98.1% and 39.8% patients on ≥ 5 and ≥ 10 drugs, respectively (from *n* = 698); women received more non-cardiovascular drugs than men (median (IQR) 3 (1–5) vs 2 (1–4), *p* = 0.014). Overall, 98.7% patients had at least one actionable genotype. Within the interaction cohort, 882 interactions were identified in 503 patients (77.1%), of which 346 in 252 patients (38.7%) were substantial: 59.2%, 11.6%, 26.3%, and 2.9% substantial interactions were DDIs, DGIs, DDGIs, and DGGIs, respectively. CYP2C19 (49.5% of all interactions) and SLCO1B1 (18.4%) were involved in the largest number of interactions. Multimorbidity (*p* = 0.019) and number of drugs (*p* = 9.8 × 10^−10^) were both associated with patients having ≥ 1 substantial interaction. Multimorbidity (HR 1.76, 95% CI 1.10–2.82, *p* = 0.019), number of drugs (HR 1.10, 95% CI 1.04–1.16, *p* = 1.2 × 10^−3^), and age (HR 1.05, 95% CI 1.03–1.07, *p* = 8.9 × 10^−7^), but not drug interactions, were associated with increased subsequent major adverse cardiovascular events.

**Conclusions:**

Multimorbidity, polypharmacy, and drug interactions are common after a NSTE-ACS. Replication of results is required; however, the high prevalence of DDGIs suggests integrating co-medications with genetic data will improve medicines optimisation.

**Supplementary information:**

**Supplementary information** accompanies this paper at 10.1186/s12916-020-01827-z.

## Background

Coronary heart disease (CHD) is a leading cause of mortality worldwide [1]. The age-adjusted CHD death rate is falling in the developed world [2, 3] leading to a higher proportion of individuals living with CHD; approximately half of this decrease is attributable to improvements in interventional and pharmacological strategies, and the other half to attenuation of risk factors [2]. Additionally, the management of multiple other conditions has improved. Thus, the number of CHD patients with multimorbidity, often defined as the co-existence of two or more chronic diseases [4], is increasing [5].

Multimorbidity increases the prevalence of polypharmacy, which some have defined as five or more medications daily [6]. Polypharmacy however can be appropriate; for instance, after acute coronary syndrome (ACS), guidelines generally recommend dual antiplatelet therapy (aspirin and a P2Y_12_ inhibitor), a high intensity statin, an angiotensin-converting enzyme inhibitor (ACEI) or angiotensin II receptor blocker (ARB), and a beta blocker [7]. However, multimorbidity often further increases the number of concomitant drugs due to treatment of related conditions, such as ACS risk factors (e.g. hypertension) and sequelae (e.g. heart failure), and treatment of other co-existing diseases. As the number of co-prescribed medications rises, the risk of drug-drug interactions (DDIs) [8, 9] and adverse drug reactions [10] increases.

Pharmacogenomics is the study and application of the genomic determinants of drug response. Therapeutic recommendations (e.g. dose or drug selection) for ‘actionable’ drug-gene interactions (DGIs) involving germline genotypes have been developed by international guideline committees for over 75 drugs, which include cardiovascular drugs and many other commonly prescribed medications [11]. Some drugs, such as warfarin, have recommendations involving more than one gene leading to drug-gene-gene interactions (DGGIs). Several large pharmacogenomics clinical implementation programmes are underway [12]; for example, the Ubiquitous Pharmacogenomics implementation study has recruited ⁓ 7000 patients from seven European sites [12]. Therefore, although pharmacogenomics is not yet commonplace, it is expected to become increasingly available in clinical practice in the coming years.

**Fig. 1:**
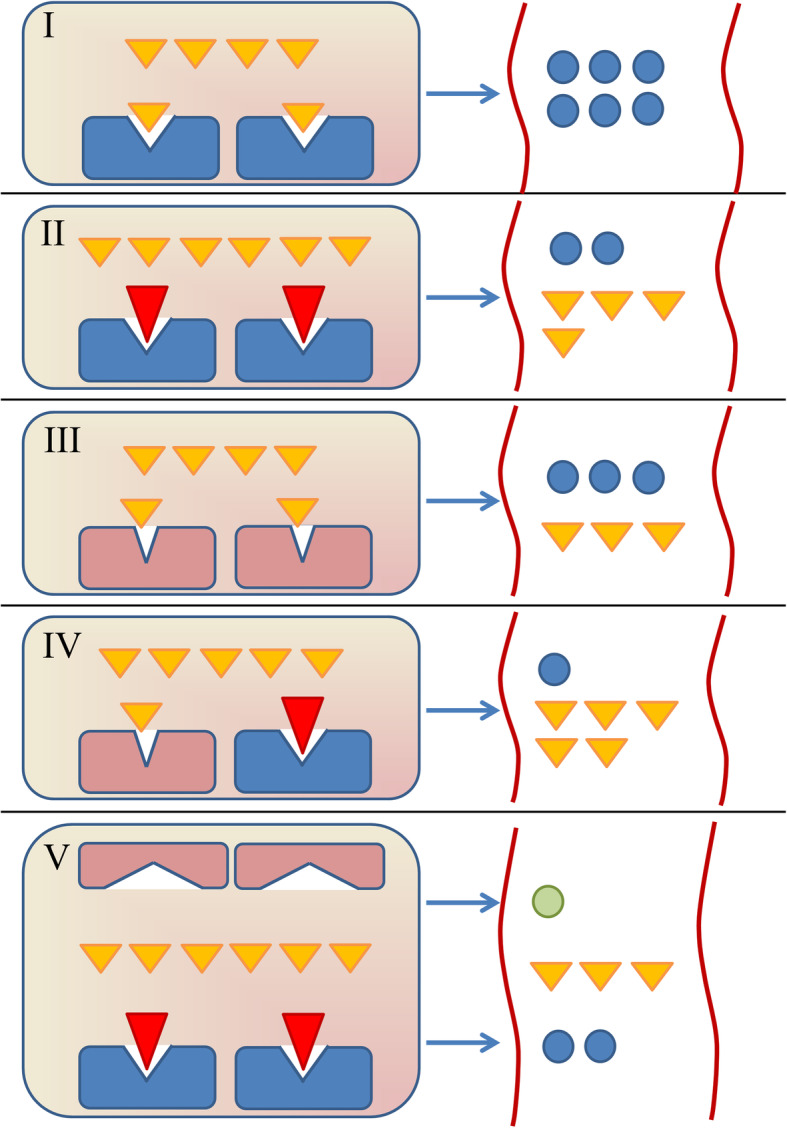
Drug-drug, drug-gene, and drug-drug-gene interactions. The hepatic extensive (normal) metabolism of small molecule drug A (yellow triangles) requires functional drug-metabolising enzyme Z (DME-Z, blue rectangles) for metabolism to metabolite A (blue circles), as shown in **I**. In **II**, DME-Z is inhibited by perpetrator drug B (red triangle) leading to reduced drug A metabolism, and hence a drug-drug interaction (DDI). Similarly in **III**, the function of DME-Z is reduced by genetic variation (red rectangles) conferring a poor metaboliser (homozygous) genotype, leading to reduced drug A metabolism, and hence a drug-gene interaction (DGI). In **IV**, a drug-drug-gene interaction (DDGI) is depicted whereby both genetic variation (e.g. a heterozygous intermediate metaboliser genotype) and perpetrator drug B conceivably act together to collectively reduce DME-Z function, strongly attenuating drug A metabolism. In **V**, drug A is primarily metabolised by DME-Z, but also a second enzyme, DME-Y, (flattened red rectangles) involved to a lesser extent is shown. If perpetrator drug B inhibits DME-Z, less drug A is metabolised by DME-Z and so drug A metabolism is more reliant on DME-Y conversion to metabolite B (green circle). However, if the genotype of DME-Y confers a reduced function (poor metaboliser DME-Y phenotype depicted in **V**), then the overall metabolism of drug A will be greatly reduced, constituting a DDGI. Similarly, if the functions of both DME-Z and DME-Y were affected by genetic variants, a drug-gene-gene interaction (DGGI) would manifest. The result of all of these interactions is less metabolism and so increased systemic exposure to drug A. These interactions equally apply to perpetrator drug inducers and genetic variation conferring rapid/ultra-rapid metaboliser predicted phenotypes. Similarly, these interactions apply to both deactivating metabolism of active drugs and bioactivation of prodrugs. Beyond enzymes, pharmacokinetic interactions can also be mediated by drug- and/or gene-based alterations to drug transporters

Interactions can primarily affect a drug’s pharmacokinetics or pharmacodynamics. Many pharmacokinetic-based interactions arise through the altered function of drug-metabolising enzymes (e.g. phase I cytochrome P450 enzymes (CYPs)) and transporters (e.g. P-glycoprotein (P-gp) encoded by *ABCB1*, and organic anion-transporting polypeptide 1B1 (OATP1B1) encoded by *SLCO1B1*). Interestingly, around half of the 200 most commonly used drugs undergo CYP-mediated metabolism [13] with CYP3A4/5, CYP2D6, CYP2C9, CYP2B6, and CYP2C19 responsible for most of this xenobiotic metabolism [14]. Importantly, the function of these enzymes and transporters can be significantly influenced by both interacting drugs [15, 16] and common genetic variants [11]. Failure to consider both factors, independently and combined, can lead to imprecise predictions regarding the function of drug-metabolising enzymes, transporters, and pharmacodynamic targets [17]. This may be one reason why it is currently challenging to predict which patients will be clinically affected by a DDI and its severity. Therefore, assessment of how DDIs are affected by genetic variation in one or more metabolic pathways (Fig. 1) is an important area to investigate to a have fuller understanding of potential adverse clinical consequences. However, drug- and gene-based interactions have only been studied together in real-world patients in a handful of studies [18–20], and no studies have focused on patients at the point of hospital discharge nor following a non-ST elevation ACS (NSTE-ACS).

Therefore, the aim of this work was to determine the extent of multimorbidity, medication use, and drug- and gene-based mainly pharmacokinetic interactions in patients discharged after a NSTE-ACS, to explore the influence of patient age and sex on these factors, and to investigate the impact of these factors on subsequent clinical events.

## Methods

### Participants

This investigation utilised the Pharmacogenetics of Acute Coronary Syndrome (PhACS) study, which has been described previously [21]. Briefly, PhACS was a UK 16-site prospective observational study between 2008 and 2013 that recruited 1470 patients hospitalised with an NSTE-ACS. Standard data collection included medical history and cardiovascular medications at discharge; other medications were optionally recorded in an open-ended medication appendix. Participants were followed up for incident events at 1 and 12 months, and the study ended when all had received 12 months follow-up. However, whilst the study was running, participants recruited earlier were followed up annually after their 12-month visit. The protocol was approved by the Liverpool UK (adult) research Ethics Committee (07/H1005/117), site-specific approval was granted at all study sites, and written informed consent was ascertained from all study subjects in accordance with the Declaration of Helsinki.

**Fig. 2:**
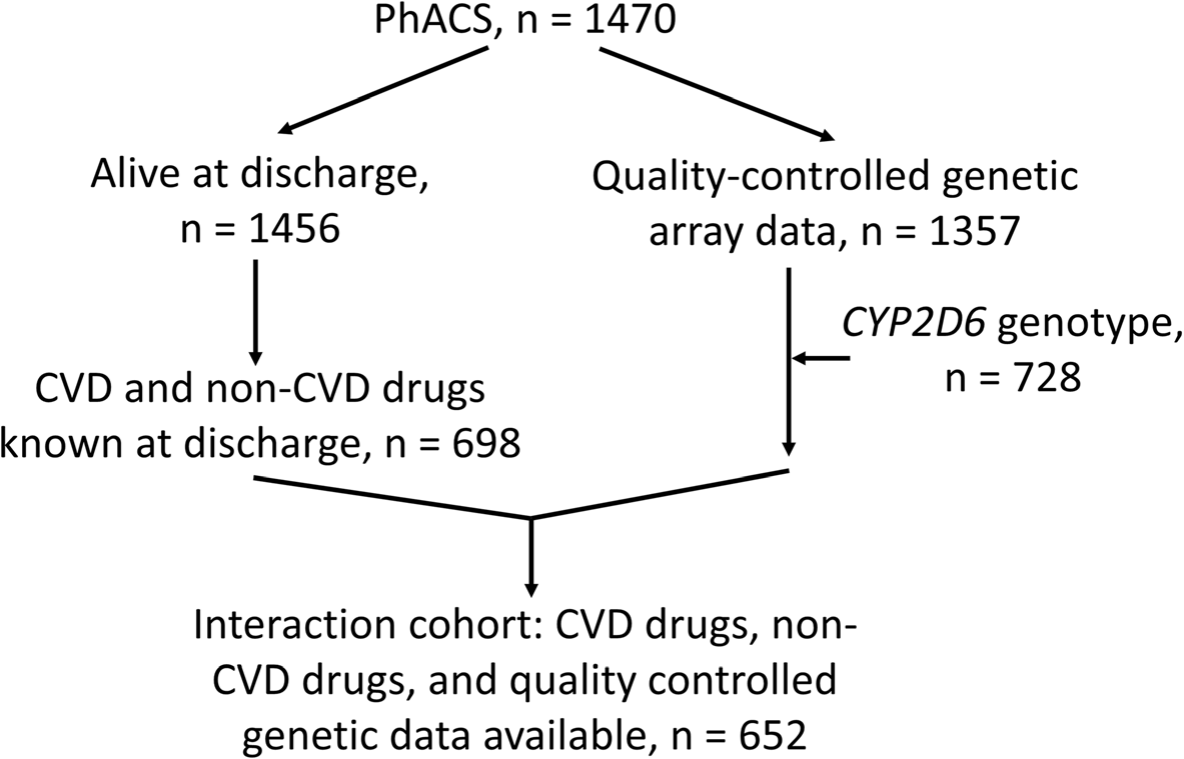
Study cohort selection process. There were 1470 patients included in the PhACS study, of whom 1456 were discharged alive from their index NSTE-ACS; 1357 had array genetic data available following standard quality control procedures; *CYP2D6* genotype was available in 728 after determination of copy number variation in those on CYP2D6 substrates; 698 had non-cardiovascular disease (CVD) drugs at discharge recorded in their case report form (CRF) medication appendix, in addition to CVD drugs explicitly required in the CRF; and in 652 patients (the interaction cohort), CVD drugs, non-CVD drugs, and genetic data were available (although patients not on a CYP2D6 substrate did not require *CYP2D6* genotype for inclusion in the interaction cohort)

In this present study, all PhACS patients alive at discharge from their index hospitalisation (*n* = 1456) were used to describe clinical characteristics and secondary prevention cardiovascular drug use, and all with available genetic data were used to assess the frequency of actionable genetic variants in pharmacogenes. Patients whose non-cardiovascular and cardiovascular drugs were both known at discharge were used to describe total drug use (*n* = 698), and of these patients, those who had quality-controlled (QC) genetic data were used to assess all drug and gene interactions (*n* = 652, the ‘interaction cohort’) (Fig. 2).

### Clinical characteristics, multimorbidity, and medication use

Within the whole cohort (*n* = 1456), sex, age, body mass index (BMI), smoking, pre-existing comorbidities, raised troponin at index NSTE-ACS, investigation of index NSTE-ACS by coronary catheterisation and treatment by percutaneous coronary intervention or coronary artery bypass grafting (PCI/CABG), and use of secondary prevention drugs (aspirin, P2Y_12_ inhibitor, ACEI/ARB, beta blocker, statin) at discharge were determined. Comorbidities were further subdivided into cardiovascular and non-cardiovascular conditions. Pre-existing cardiovascular multimorbidity was defined as ≥ 2 of the following: hypertension, prior myocardial infarction (MI, did not include index NSTE-ACS), stroke, transient ischaemic attack, subarachnoid haemorrhage, and peripheral artery disease. Pre-existing non-cardiovascular multimorbidity was ≥ 2 of the following: chronic pulmonary disease (chronic obstructive pulmonary disease, contemporary asthma, bronchiectasis, pulmonary fibrosis), diabetes, chronic kidney disease (CKD, serum creatinine > 150 μmol/L) or renal transplantation, peptic ulcer disease, chronic liver disease (alcoholic liver disease, cirrhosis, autoimmune hepatitis), osteoarthritis, connective tissue disease (e.g. rheumatoid arthritis, polymyalgia rheumatica), chronic neurological conditions (e.g. epilepsy, motor neuron disease, dementia, Parkinson’s disease), and any cancer. Only physical illnesses were recorded in PhACS; mental health disease data were not available. Overall multimorbidity was defined as ≥ 2 of any of the above cardiovascular and non-cardiovascular conditions, in keeping with the Academy of Medical Sciences definition [22].

The individually recorded discharge medications in those with complete drug data (*n* = 698) were grouped together according to the British National Formulary chapter-level categories [23]. However, drugs in the skin, eye, and ear/nose/oropharynx chapters were combined here due to being mainly non-oral. An ‘Other’ category was also constructed, consisting mainly of small numbers of drugs from the malignant disease/immunosuppression and gynaecology/urinary tract disorder chapters. A patient was counted for a category if they were on one or more drugs within that category.

### Identification of actionable genetic variants

Participants were genotyped using the Illumina HumanOmniExpressExome-8 v1.0 BeadChip at Edinburgh Genomics (Roslin Institute, Scotland). The genetic QC and imputation for PhACS have been described previously, with 1357 patients passing all QC [24]. The following actionable variants were extracted: CYP2B6*6/*9 (rs3745274), CYP2C9*2 (rs1799853), *3 (rs1057910), CYP2C19*2 (rs4244285), *17 (rs12248560), CYP3A5*3 (rs776746), CYP2D6*3 (rs35742686), *4 (rs3892097), *9 (rs5030656), *10 (rs1065852), *41 (rs28371725), *DPYD* HapB3 (rs56038477), *F5* (rs6025), SLCO1B1*5/*15 (rs4149056), TPMT*3B (rs1800460), *3C (rs1142345), *3A (rs1800460 with rs1142345), *UGT1A1* -364C>T (rs887829), and *VKORC1* 1173C>T (rs9934438). The variant allele of *UGT1A1* rs887829 is in linkage disequilibrium (*r*^2^ ≈ 0.99) with the tandem nucleotide repeat polymorphism (UGT1A1*28) [25]. Of these 18 variants, 10 contained imputed values (information scores all > 0.7, indicating good imputation quality). If required, imputed genotypes were categorised as wild-type (imputed score 0–0.3), heterozygous (0.7–1.3), and homozygous (1.7–2).

*CYP2D6* copy number variants (CNVs) were identified with the TaqMan™ copy number assay (Thermo Fisher Scientific) in those on *CYP2D6* substrate drugs (*n* = 728). qPCR data were acquired using Quantstudio 6 Flex Real-Time PCR System (Thermo Fisher Scientific) and analysed with CopyCaller v2.1 software (Life Technologies). *CYP2D6* genotype to metaboliser phenotype translation was based on recent consensus recommendations from the Clinical Pharmacogenetics Implementation Consortium (CPIC) and Dutch Pharmacogenetics Working Group (DPWG) [26], determined from CYP2D6 activity score (AS) as follows: poor metaboliser (PM, AS = 0), intermediate metaboliser (IM, AS = 0.25–1), extensive metaboliser (EM, AS = 1.25–2.25), and ultra-rapid metaboliser (UM, AS > 2.25). The metaboliser phenotypes for the other *CYPs* were determined from CPIC standard diplotype-phenotype tables [11].

### Drug substrates, inhibitors, and inducers for drug-drug interactions

Drug substrates, inhibitors, and inducers for CYP1A2, CYP2B6, CYP2C8, CYP2C9, CYP2C19, CYP2D6, and CYP3A4/5 are listed in Additional files 1, 2, and 3 [15, 16, 27] and were based on the US Food and Drug Administration (FDA) clinical tables [15] and the Indiana University cytochrome P450 drug interactions Flockhart Table™ [16] (both accessed January 2019), with specific input from the literature when required [27]. Tobacco smoke constituted a CYP1A2 inducer.

Substrates and inhibitors for drug transporters, P-gp and SLCO1B1, are in Additional file 4 [15, 28–30]. Transporter substrates/inhibitors were extracted from the FDA clinical tables [15] with supplementation of P-gp substrates and addition of P-gp inducers from Wessler et al. [28]; no SLCO1B1 inducers were listed. Assessment of the strength of transporter inhibitors/inducers was based on relevant literature [28–30].

A DDI was present if a patient was on a victim drug and perpetrator drug that influenced the victim drug through one or more investigated enzymes/transporters; autoinhibition/autoinduction was not considered interactions.

### Drug substrates for drug-gene interactions

For DGIs, substrate drugs were limited to those with a pharmacogenomic clinical guideline available from CPIC/DPWG for the above genotyped pharmacogenes (Additional file 5) [11, 31–41]. For each substrate, only the genotypes/metaboliser statuses considered actionable for that drug contributed to a DGI; thus, for example, *CYP2D6* IM was considered a DGI with amitriptyline but not codeine.

### Assessment of drug and gene interactions

We considered pharmacokinetic drug- and gene-based interactions and two pharmacodynamic interactions: warfarin-*VKORC1* rs9934438 (affects warfarin dose) and oestrogen-containing contraceptives-*F5* rs6025 (augments the risk of venous thromboembolism).

First, we identified and counted the total number of unique DDIs, DGIs, drug-drug-gene interactions (DDGIs), and DGGIs of any strength (‘all’ interactions). Second, we identified those interactions predicted to have a ‘substantial’ effect, defined as follows:

i) DDIs involving strong drug inhibitors/inducers. Strong CYP inhibitors increase the area under the concentration-time curve (AUC) of sensitive substrates ≥ 5-fold, strong CYP inducers decrease the AUC of sensitive substrates by ≥ 80%, and strong transporter inhibitors/inducers were determined from the literature [28–30].
ii) DGIs due to variant homozygous/compound heterozygous genotypes.
iii) DDGIs due to any strength perpetrator drug and actionable genotype provided the directions of effect of the constituent DDI and DGI on the victim drug were the *same* (i.e. all interactions would be expected to increase/decrease victim drug exposure/response). The same principle was applied to DGGIs.

The numbers of interactions per patient and gene were determined. Unless otherwise stated, each interaction in a given patient was only counted once. Thus, for example, a DDGI was counted as one DDGI, whilst its constituent DDI and DGI were not counted.

### Clinical endpoints

The main endpoints of PhACS were major adverse cardiovascular events (MACE—a composite of cardiovascular death and non-fatal MI or ischaemic stroke) and all-cause mortality (ACM) [21]. It was expected that interactions influencing cardiovascular drugs would predominate given PhACS is a cardiovascular cohort. Thus, these available main endpoints were harnessed to explore whether interactions and other relevant factors (see below) might be associated with clinical sequelae. A calculation of power was not conducted as this assessment was exploratory using available events.

### Statistical analysis

Categorical clinical variables are presented as group number (percentage), age as mean (standard deviation) as normally distributed, and BMI as median (interquartile range (IQR) as non-normally distributed; integer count variables are presented as median (IQR/range) for numbers of drugs and interactions, or number of all comorbidities (percentage).

To assess for baseline differences between the interaction cohort and the remainder of the whole cohort, the following were compared: clinical characteristics and secondary prevention drug use (Pearson’s chi-squared test), number of comorbidities and overall number of cardiovascular drugs (Fisher’s exact test), age (Student’s *t* test), and BMI (Mann-Whitney *U* test).

In the whole cohort, age was associated with patient sex (*p* = 2.0 × 10^−8^). Thus, the influence of sex and age on clinical variables/drug use was ascertained by their inclusion in appropriate regression (linear/logistic/Poisson/negative binomial) models. In all analyses involving age, when a quadratic relationship with an outcome was suspected from visual inspection, age and age squared were entered into the model. In this situation, age was mean centred before its squared term calculated. When a linear relationship was identified, uncentred age was used.

To investigate the association between multimorbidity and number of drugs, Poisson regression was used, whilst logistic regression assessed whether multimorbidity was associated with the number of patients that received all five secondary prevention cardiovascular drug classes (vs < 5); both analyses were adjusted for age.

Multicollinearity was tested between age (or with mean centred age and age squared), sex, multimorbidity, number of drugs, and all and substantial interactions and was not found (highest variance inflation factor < 1.73). Thus, age, sex, multimorbidity, and number of drugs underwent univariate and multivariable logistic regression with forward (likelihood ratio) variable selection to investigate associations with patients that have ≥ 1 interaction, or ≥ 1 substantial interaction (vs patients with < 1). Lastly, univariate and multivariable Cox’s proportional hazards regression tested whether age, sex, multimorbidity, number of drugs, and all or substantial interactions were associated with time to incident MACE, or ACM, during follow-up within the interaction cohort.

In sensitivity analyses, dichotomous multimorbidity was replaced by number of comorbidities and all relevant analyses repeated. *p* < 0.05 was taken as statistically significant as all analyses were exploratory. Statistical analysis was carried out in the R computing environment [42] version 3.5.1 or above and IBM SPSS version 24.0 (IBM Corp, Armonk, NY, USA).

## Results

### Baseline differences in cohorts

Table 1 reports baseline differences between the interaction cohort (*n* = 652) and other patients from the whole cohort (*n* = 804). The interaction cohort was marginally older (*p* = 0.012) and included more women (*p* = 0.040). The main difference was the higher prevalence of multimorbidity in the interaction cohort (59.0% vs 42.5%, *p* = 3.7 × 10^−10^).

**Table 1:** Comparison of those included and not included in the interaction cohort

Characteristic	Interaction cohort, ***n*** = 652	Not in interaction cohort, ***n*** = 804	Unadjusted ***p*** value
**Demographics**			
Sex (male), *n* (%)	457 (70.1)	600 (74.6)	0.040
Age (years), mean (SD)	66.0 (11.5)	64.4 (11.8)	0.012
BMI, median (IQR)	28.3 (25.3–31.8)	27.9 (24.9–31.4)	0.21
**Comorbidities**			
Smoking, *n* (%)	185 (28.4)	180 (22.4)	0.011
Cardiovascular multimorbidity	218 (33.4)	175 (21.8)	6.1 × 10^−7^
Non-cardiovascular multimorbidity	167 (25.6)	98 (12.2)	4.1 × 10^−11^
All multimorbidity	385 (59.0)	342 (42.5)	3.7 × 10^−10^
Number of comorbidities, *n* (%)			
0	116 (17.8)	205 (25.5)	5.0 × 10^−4^
1	151 (23.2)	257 (32.0)	
2	152 (23.3)	174 (21.6)	
3	107 (16.4)	105 (13.1)	
4–5	117 (17.9)	55 (6.8)	
6–7	9 (1.4)	8 (1.0)	
**Index NSTE-ACS**			
Raised troponin, *n* (%)	623 (95.6)	765 (95.1)	0.45
Coronary catheterisation, *n* (%)	445 (68.3)	529 (65.8)	0.32
PCI/CABG, *n* (%)	283 (43.4)	376 (46.8)	0.20
**Cardiovascular drugs at discharge**			
Aspirin, *n* (%)	612 (93.9)	758 (94.3)	0.52
P2Y_12_ inhibitor, *n* (%)	556 (85.3)	680 (84.6)	0.81
ACEI/ARB, *n* (%)	540 (82.8)	651 (81.0)	0.39
Beta blocker, *n* (%)	528 (81.0)	664 (82.6)	0.34
Statin, *n* (%)	609 (93.4)	767 (95.4)	0.026
Number of patients on all five secondary prevention cardiovascular drugs, *n* (%)^†^	373 (57.2)	464 (57.7)	0.84
Number of cardiovascular drugs/patient, median (IQR, range)^††^	6 (5–7, 0–9)	6 (5–6, 2–10)	0.11

*ACEI* angiotensin-converting enzyme inhibitor, *ARB* angiotensin II receptor blocker, *CABG* coronary artery bypass graft surgery, *IQR* interquartile range, *n (%)* number (percent) of patients, *PCI* percutaneous coronary intervention, *SD* standard deviation

^†^Secondary prevention cardiovascular drugs were aspirin, P2Y_12_ inhibitor, ACEI/ARB, beta blocker, and a statin

^††^Includes secondary prevention and other cardiovascular drugs

### Clinical variables, comorbidities, medication use, and influence of patient age and sex

Patient clinical characteristics and medication use are shown in Table 2. In the whole cohort (*n* = 1456), 72.6% were male, mean age was 65.1 years old (standard deviation 11.7, range 26.4–93.4), 26.9% had cardiovascular multimorbidity, 18.1% had non-cardiovascular multimorbidity (most commonly diabetes, then osteoarthritis and chronic pulmonary diseases), 49.9% had ≥ 2 any comorbidities, and 4.7% had ≥ 5 any comorbidities. The index NSTE-ACS was treated with PCI/CABG in 45.3% patients, and 57.5% received a drug from all five secondary prevention cardiovascular drug classes at discharge. In those with full drug data (*n* = 698), 98.1%, 39.8%, and 6.2% were on at least five, ten, and 15 different drugs, respectively; the median numbers of cardiovascular, non-cardiovascular, and total drugs per patient were 6 (IQR 5–7, range 0–9), 2 (IQR 1–4, range 0–16), and 9 (IQR 7–11, range 2–26), respectively.

**Table 2:** Clinical characteristics, multimorbidity, and medication use

Characteristic	Female, ***n*** = 396	Male, ***n*** = 1057	Adjusted ***p*** value^†^	All, ***n*** = 1456^††^
**Demographics**				
Age (years), mean (SD)	68.0 (11.6)	64.1 (11.6)	2.0 × 10^−8^	65.1 (11.7)
BMI, median (IQR)	27.5 (24.2–32.5)	28.3 (25.4–31.5)	0.85	28.1 (25.0–31.6)
**Comorbidities**				
Smoking, *n* (%)	98 (24.7)	266 (25.2)	0.060	365 (25.1)
Cardiovascular multimorbidity, *n* (%)	104 (26.2)	288 (27.2)	0.12	392 (26.9)
Non-cardiovascular multimorbidity, *n* (%)	96 (24.2)	168 (15.9)	8.0 × 10^−3^	264 (18.1)
All multimorbidity, *n* (%)^†††^	217 (54.8)	507 (48.0)	0.39	727 (49.9)
Number of comorbidities, *n* (%)^†††^				
0	77 (19.4)	244 (23.1)	0.60	321 (22.0)
1	102 (25.8)	306 (28.9)		408 (28.0)
2	97 (24.5)	227 (21.5)		326 (22.4)
3	56 (14.1)	155 (14.7)		212 (14.6)
4–5	59 (14.9)	113 (10.7)		172 (11.8)
6–7	5 (1.3)	12 (1.1)		17 (1.2)
**Index NSTE-ACS**				
Raised troponin, *n* (%)	378 (95.5)	1007 (95.3)	0.91	1388 (95.3)
Coronary catheterisation, *n* (%)	250 (63.1)	723 (68.4)	0.49	974 (66.9)
PCI/CABG, *n* (%)	156 (39.4)	502 (47.5)	0.082	659 (45.3)
**Cardiovascular drugs at discharge**				
Aspirin, *n* (%)	371 (93.7)	996 (94.2)	0.82	1370 (94.1)
P2Y_12_ inhibitor, *n* (%)	334 (84.3)	899 (85.1)	0.89	1236 (84.9)
ACEI/ARB, *n* (%)	318 (80.3)	871 (82.4)	0.61	1191 (81.8)
Beta blocker, *n* (%)	300 (75.8)	889 (84.1)	1.8 × 10^−3^	1192 (81.9)
Statin, *n* (%)	367 (92.7)	1006 (95.2)	0.14	1376 (94.5)
Patients on all five secondary prevention cardiovascular drugs, *n* (%)^‡^	205 (51.8)	630 (59.6)	0.081	837 (57.5)
**Drug use by category at discharge**				
***N*** **= 698**^‡‡^	***n*** **= 208**	***n*** **= 490**		***n*** **= 698**
Gastro-intestinal, *n* (%)	129 (62.0)	267 (54.5)	0.18	396 (56.7)
Cardiovascular, *n* (%)	207 (99.5)	490 (100.0)	0.99	697 (99.9)
Respiratory, *n* (%)	45 (21.6)	99 (20.2)	0.85	144 (20.6)
Central nervous system, *n* (%)	85 (40.9)	160 (32.7)	0.026	245 (35.1)
Infections, *n* (%)	17 (8.2)	33 (6.7)	0.65	50 (7.2)
Endocrine, *n* (%)	83 (39.9)	136 (27.8)	0.012	219 (31.4)
Nutrition and blood, *n* (%)	44 (21.2)	63 (12.9)	0.024	107 (15.3)
Musculoskeletal, *n* (%)	36 (17.3)	65 (13.3)	0.37	101 (14.5)
Eye, ear, nose, oropharynx, and skin, *n* (%)	10 (4.8)	28 (5.7)	0.33	38 (5.4)
Other, *n* (%)	9 (4.3)	41 (8.4)	0.017	50 (7.2)
**Numbers of drugs at discharge**				
Number of cardiovascular drugs/patient, median (IQR, range)	6 (5–6, 0–9)	6 (5–7, 2–9)	0.21	6 (5–7,0–9)
Number of non-cardiovascular drugs/patient, median (IQR, range)	3 (1–5, 0–15)	2 (1–4, 0–16)	0.014	2 (1–4, 0–16)
Number of drugs/patient, median (IQR, range)	9 (8–11, 2–22)	9 (7–11, 3–26)	0.20	9 (7–11, 2–26)

*ACEI* angiotensin-converting enzyme inhibitor, *ARB* angiotensin II receptor blocker, *CABG* coronary artery bypass graft surgery, *IQR* interquartile range, *n (%)* number (percent) of patients, *PCI* percutaneous coronary intervention, *SD* standard deviation

^†^Adjusted for age, except when testing the association between sex and age itself

^††^The sex of three patients was missing from the *n* = 1456 cohort; thus, these patients were not included in the sex-stratified columns, but were counted in the All column

^†††^The age-adjusted *p* value for the association between sex and all comorbidities was 0.39 when comorbidities were dichotomised into multimorbidity (≥ 2 vs < 2 any cardiovascular or non-cardiovascular conditions) and *p* = 0.60 when number of comorbidities was treated as an integer count within negative binomial regression

^‡^Secondary prevention cardiovascular drugs were aspirin, P2Y_12_ inhibitor, ACEI/ARB, beta blocker, and a statin

^‡‡^For drug categories within the *n* = 698 cohort, a patient was counted if they were on one or more drugs within a given category

As expected, increasing age was strongly associated with increased multimorbidity (*p* = 1.8 × 10^−22^) and higher total drug use (*p* = 2.1 × 10^−8^). Increasing age was also associated with fewer patients currently smoking (*p* = 6.0 × 10^−35^) or receiving drugs from all five secondary prevention drug classes (*p* = 1.5 × 10^−8^) (see Additional file 6, which additionally includes results if age dichotomised at 65). Interestingly, non-linear relationships were observed between age and number of comorbidities (a decline in the rate of acquiring comorbidities at older age), and especially with both coronary catheterisation and PCI/CABG for the index NSTE-ACS (a decrease in interventions at older age, see Additional file 7). No other non-linear age relationships were detected (including when investigating interactions, MACE, and ACM).

Women were almost 4 years older on average in the whole cohort (Table 2). After adjustment for age, women had more non-cardiovascular multimorbidity than men (24.2% vs 15.9%, *p* = 0.008) but there were no differences between the sexes in cardiovascular multimorbidity (*p* = 0.12) or overall number of comorbidities (*p* = 0.60). Trends were observed for fewer women receiving PCI/CABG (39.4% vs 47.5%, *p* = 0.082) or drugs from all five cardiovascular secondary prevention drug classes (51.8% vs 59.6%, *p* = 0.081) compared to men. Notably, women were prescribed more non-cardiovascular drugs than men (median (IQR) 3 (1–5) vs 2 (1–4), *p* = 0.014). Specifically, a higher proportion of women received one or more drugs in the central nervous system category (40.9% vs 32.7%, *p* = 0.026, mainly antidepressants and analgesics), endocrine system category (39.9% vs 27.8%, *p* = 0.012, a combination including more insulin, levothyroxine, bisphosphonates, and oestrogens/progestrogens), and nutrition and blood category (21.2% vs 12.9%, *p* = 0.024; mainly iron supplementation and vitamin replacement) compared to men. Overall though, total drug use did not differ between women and men (median (IQR) of 9 (8–11) vs 9 (7–11), *p* = 0.20).

All multimorbidity (≥ 2 conditions vs < 2), adjusted for age, was strongly associated with both increased total number of drugs/patient (median (IQR) 10 (8–12) vs 8 (7–9), *p* < 2.0 × 10^−16^) and less patients being prescribed all five secondary prevention cardiovascular drug classes (51.4% vs 64.9%, respectively, *p* = 1.7 × 10^−4^). These associations persisted when multimorbidity was stratified into cardiovascular and non-cardiovascular multimorbidity, although non-cardiovascular multimorbidity was particularly strongly associated with fewer patients receiving all five secondary prevention drug classes (39.2% vs 62.4%, age-adjusted *p* = 5.8 × 10^−9^).

### Patient genotypes

**Fig. 3:**
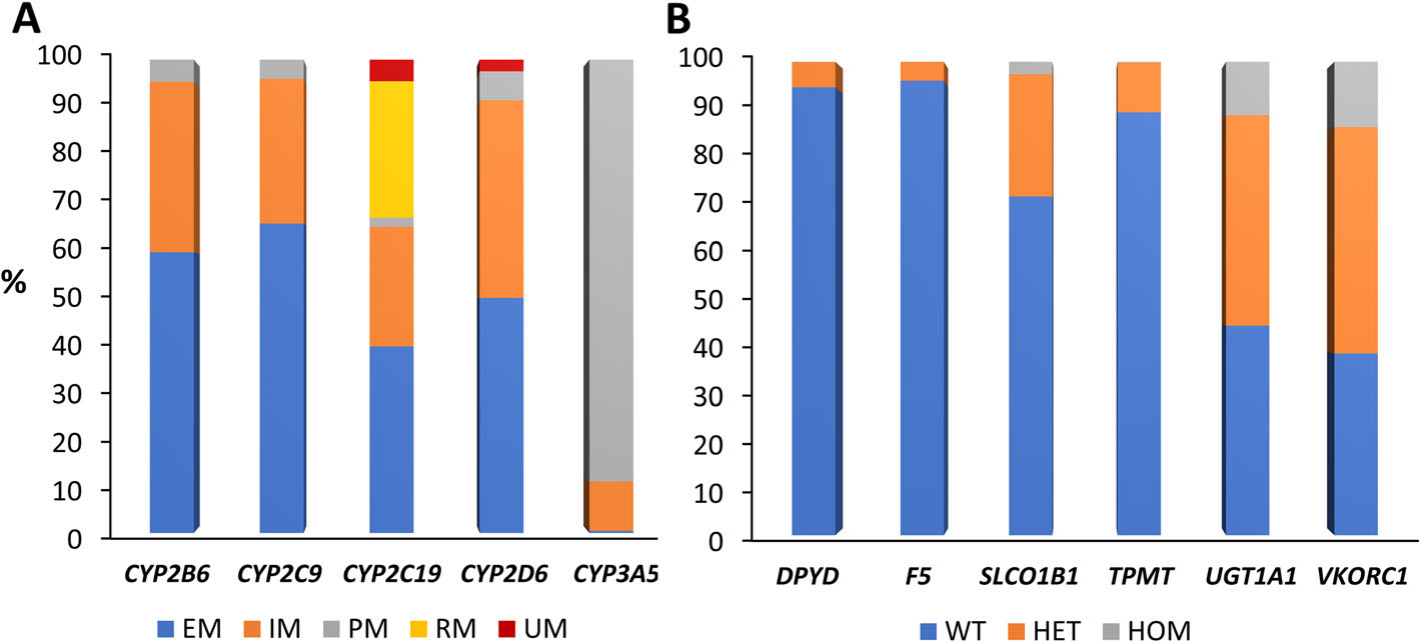
Patient pharmacogene genotypes. Proportions of genotype-based *CYP* metaboliser phenotypes (**a**) and non-*CYP* pharmacogene genotypes (**b**) observed across the whole cohort with quality-controlled array genetic data available (*n* = 1357), except for *CYP2B6* (*n* = 1347), *F5* (*n* = 1346), and *CYP2D6* (*n* = 728). The number of patients with *CYP2B6*, *F5*, and *CYP2D6* available was less due to exclusion of patients with imputed genotypes outside the predefined imputation acceptance range, and determination of *CYP2D6* copy number variation in patients on a CYP2D6 drug substrate (see the ‘Methods’ section). Overall, 713 patients had genotypes available for all analysed genes. EM, extensive (normal) metaboliser; IM, intermediate metaboliser; PM, poor metaboliser; RM, rapid metaboliser; UM, ultra-rapid metaboliser; WT, wild-type; HET, heterozygous; HOM, homozygous for the variant allele

Figure 3 shows patient genotype-based metaboliser phenotypes (*CYPs*) and genotypes (non-*CYPs*) for actionable pharmacogenes. Importantly, 98.7% of patients with genotypes available for all analysed genes (*n* = 713 inclusive of *CYP2D6* CNVs) had at least one actionable genotype (range 0–7 actionable genotypes from 11 genes).

### Drug and gene interactions

In the interaction cohort (*n* = 652), the overall numbers of different drug substrates, inhibitors, and inducers considered were 199, 109, and 27, respectively. Twenty-four drugs with an associated pharmacogenomic guideline were prescribed in the interaction cohort, 17 of which contributed to ≥ 1 observed DGI (Additional file 5). The genes with DGIs were as follows: *CYP2C9*, *CYP2C19*, *CYP2D6*, *CYP3A5*, *SLCO1B1*, and *VKORC1*.

**Fig. 4:**
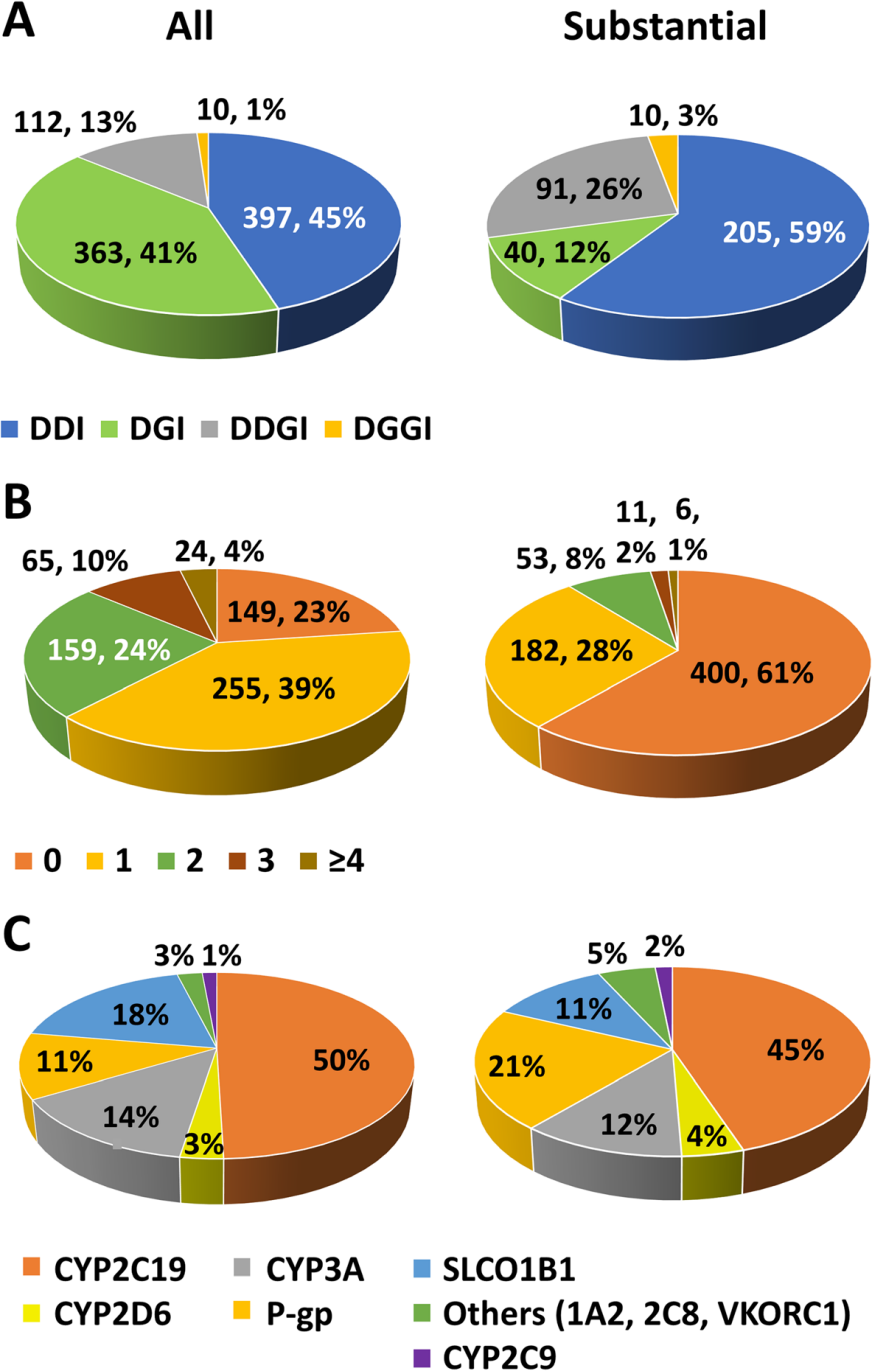
Drug- and gene-mediated interactions. This figure shows the total number (%) of interactions identified (**a**), the number (%) of interactions per patient (**b**), and the percentage of interactions mediated per enzyme/transporter (**c**) in 652 post-NSTE-ACS patients. The pie charts on the left display all interactions (of any or unconfirmed strength), whilst predicted substantial interactions only are presented in the charts on the right. In **a** and **b**, each interaction was counted only once. In **c**, some types of interaction were counted more than once in a given patient. These were as follows: DDIs where a perpetrator drug can influence the same victim drug through more than one enzyme/transporter (e.g. verapamil inhibiting atorvastatin at both CYP3A4/5 and P-gp), DDGIs involving more than one enzyme/transporter, and DGGIs. These interactions were included in the count of both involved enzymes/transporters to illustrate them clearly in **c** only

Overall, 882 interactions in 503 (77.1%) patients were identified, whilst 346 substantial interactions occurred in 252 (38.7%) patients (Fig. 4a). Almost half (45.0%) of all interactions were DDIs with 41.2% DGIs, 12.7% DDGIs, and 1.1% DGGIs. For substantial interactions, 59.2% were DDIs, 11.6% DGIs, 26.3% DDGIs, and 2.9% DGGIs. The observed DGGIs were due to either warfarin/*CYP2C9*/*VKORC1* or amitriptyline/*CYP2D6*/*CYP2C19* interactions. A complete breakdown of all and substantial interactions is in Additional files 8 and 9, respectively.

**Fig. 5:**
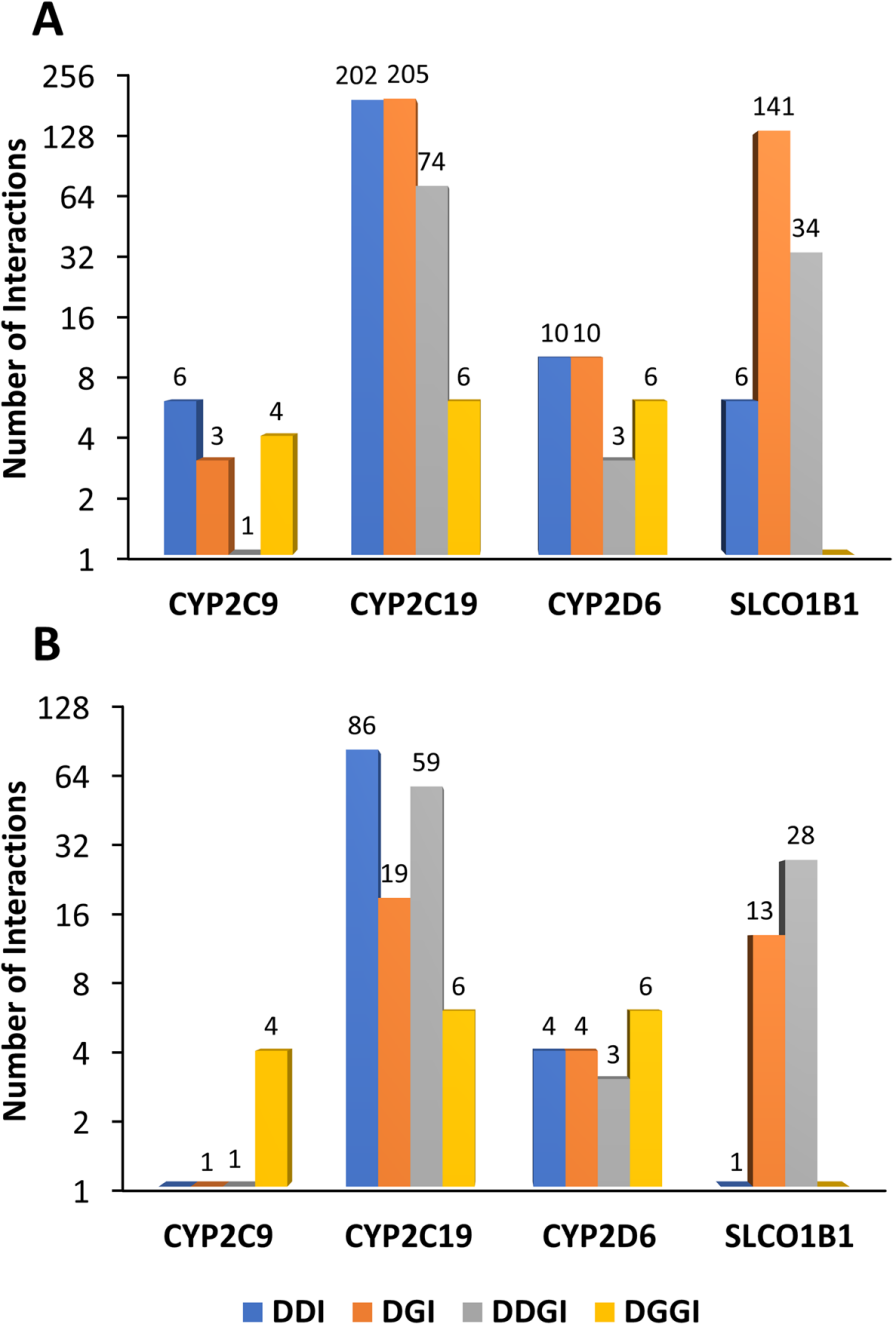
Interactions mediated by CYP2C9, CYP2C19, CYP2D6, and SLCO1B1. This figure displays the number of identified all (**a**) and substantial (**b**) interactions mediated through the three CYP enzymes (2C9, 2C19, 2D6) and transporter (SLCO1B1) that were directly influenced by both drug- and gene-based interactions, in 652 post-NSTE-ACS patients. In this patient cohort, CYP2C19-mediated interactions were the most common due to the high frequency of clopidogrel prescribing and, to a lesser extent, proton pump inhibitors, although many SLCO1B1-based interactions were also found due to atorvastatin and simvastatin

Of those with interactions, the median number (range) of all and substantial interactions per patient was 1 (1–9) and 1 (1–5), respectively (Fig. 4b). Interactions involving CYP2C19 were the most common (49.5% of all interactions), followed by SLCO1B1 (18.4%), CYP3A4/5 (13.8%), and P-gp (11.4%, Fig. 4c). Almost a third of all and substantial interactions involved transporters (P-gp, SLCO1B1, Fig. 4c). CYP2C9, CYP2C19, CYP2D6, and SLCO1B1 mediated both drug- and genotype-based interactions (Fig. 5). CYP2C19 interactions were the most common as 84.7% of patients were prescribed the CYP2C19 substrate, clopidogrel, and 44.2% a proton pump inhibitor (44.4% of these were (es)omeprazole, 52.8% lansoprazole, 2.8% other), which are CYP2C19 substrates and inhibitors. The majority of SLCO1B1 interactions were due to atorvastatin (79.9% of patients received) or, to a lesser extent, simvastatin (10.0% received). Thus, whilst DDIs made up 41.5% (202/487) of all CYP2C19-mediated interactions, 96.7% (175/181) of SLCO1B1-mediated interactions were dependent on *SLCO1B1* rs4149056 (77.9% DGIs, 18.8% DDGIs).

The median number (IQR, range) of interactions in those with and without multimorbidity was 1 (1–2, 0–9) and 1 (0–2, 0–4), respectively. Multimorbidity (*p* = 0.019) and number of drugs (*p* = 9.8 × 10^−10^) were both associated with patients that have ≥ 1 substantial interaction, and number of drugs (*p* = 3.8 × 10^−8^) with ≥ 1 all interaction, in multivariable analysis. Neither patient sex nor age was associated with all/substantial interactions (Additional file 10).

### Survival analysis

There were 114 MACE and 74 ACM during a median follow-up of 19 months from hospital discharge within the interaction cohort. In multivariable survival analysis, age (*p* = 8.9 × 10^−7^), multimorbidity (*p* = 0.019), and number of drugs (*p* = 1.2 × 10^−3^) were associated with an increased risk of MACE, and age (*p* = 1.9 × 10^−12^) and number of drugs (*p* = 4.0 × 10^−4^) with increased ACM (Table 3). Whilst number of substantial interactions was borderline associated with MACE in univariate survival analysis (*p* = 0.053), it was not associated when added to the selected multivariable model (*p* = 0.89).

**Table 3:** Associations with time to major adverse cardiovascular events and all-cause mortality

	MACE		ACM	
	HR (95% CI)	***p*** value	HR (95% CI)	***p*** value
**Univariate analysis**				
Sex (F vs M)	1.49 (1.02–2.18)	0.038	1.40 (0.88–2.24)	0.16
Age	1.05 (1.04–1.07)	2.0 × 10^−9^	1.09 (1.06–1.11)	1.9 × 10^−12^
All multimorbidity	2.69 (1.73–4.20)	1.2 × 10^−5^	2.75 (1.56–4.84)	4.8 × 10^−4^
Number of drugs	1.15 (1.10–1.21)	2.0 × 10^−8^	1.14 (1.08–1.21)	3.0 × 10^−6^
Number of all interactions	1.16 (0.995–1.34)	0.058	1.01 (0.83–1.23)	0.93
Number of substantial interactions	1.23 (0.997–1.51)	0.053	1.11 (0.84–1.46)	0.47
**Multivariable analysis**				
Age	1.05 (1.03–1.07)	8.9 × 10^−7^	1.08 (1.06–1.11)	1.9 × 10^−12^
All multimorbidity	1.76 (1.10–2.82)	0.019	–	–
Number of drugs	1.10 (1.04–1.16)	1.2 × 10^−3^	1.12 (1.05–1.19)	4.0 × 10^−4^

*ACM* all-cause mortality, *CI* confidence interval, *HR* hazard ratio, *MACE* major adverse cardiovascular events

All types of interaction (drug-drug, drug-gene, drug-drug-gene, and drug-gene-gene) were counted to determine numbers of all and numbers of predicted substantial interactions per patient

### Sensitivity analysis

When multimorbidity was replaced by number of comorbidities, all results remained equivalent, although the association between comorbidities and patients with ≥ 1 substantial interaction, adjusted for number of drugs, was reduced to borderline significance (*p* = 0.071, Additional file 11).

## Discussion

The main findings of this study are: multimorbidity, polypharmacy, and drug interactions are common post-NSTE-ACS; drug- and gene-mediated perpetrators are involved in 85.5% (59.2% DDIs, 26.3% DDGIs) and 40.8% (11.6% DGIs, 26.3% DDGIs, 2.9% DGGIs) of identified substantial interactions, respectively; and both multimorbidity and number of drugs increase the risk of substantial interactions. Importantly, older age, multimorbidity, and number of drugs were associated with increased MACE, but despite trends, no statistically significant associations were found between all/substantial interactions and MACE or ACM. Lastly, differences in drug use were observed based on patient age and sex, and increasing age was associated with reduced coronary intervention.

The prevalence of multimorbidity is increasing [5] and so recognition of its clinical impact is important. We found multimorbidity post-NSTE-ACS to be associated with increased drug use, yet a lower likelihood of being prescribed secondary prevention medications, in keeping with other studies [43, 44]. Furthermore, multimorbidity and number of drugs were both associated with an increased likelihood of predicted substantial interactions. Of note, the interaction cohort had a higher proportion of patients with multimorbidity relative to the whole cohort, likely from exclusion of patients with no drug entries in their medication appendix due to being unable to ascertain whether this was accurate or represented missing data. Nevertheless, as clopidogrel and atorvastatin were the two most common victim drugs and were prescribed to similar extents across the whole cohort, the main interaction findings remain plausibly applicable to other cardiovascular patients, although further investigation in separate cohorts to determine generalisability is warranted. Still, the interaction cohort was likely enriched for patients with extreme polypharmacy and interaction burden; conceivably, it is these patients that may benefit the most from comprehensive medicines optimisation incorporating pharmacogenomics.

Importantly, the interactions assessed here included both genetic risk variants and drug perpetrators. In fact, 98.7% of patients had at least one actionable pharmacogene. In practice, only a small proportion of identified DDIs lead to clinically significant events [45]. Thus, 5–15% of older patients might suffer clinically significant adverse reactions involving drug interactions, whereas 35–60% have an identified DDI [45]. The reasons for this are complex [46], but genetic factors likely play a role, and therefore, identification of DDGIs is plausibly important, although these remain currently a novel and under-investigated subset of interactions. Still, the proportion of substantial interactions identified as DDGIs here (26.3%) was similar to estimates (19–22%) from the two previous studies in clinically heterogeneous patient populations [18, 19]. The reason(s) for the slightly higher DDGI estimate here likely pertains to considering *SLCO1B1* as well as *CYPs*, and assessing NSTE-ACS patients enriched for multimorbidity mostly taking clopidogrel that requires CYP2C19 bioactivation.

One example of a cardiovascular DDGI is between warfarin and simvastatin, whereby simvastatin reduces warfarin dose requirements by 25% and 43% in CYP2C9*3 heterozygous and homozygous patients, respectively [47]. Nevertheless, a quantitative estimate of the impact of most DDGIs on victim drug systemic exposure is currently unavailable. A general principle appears to be that drug inhibitors decrease the metabolism of victim drugs to a greater extent in *CYP* EMs and IMs compared to PMs [48]. Nevertheless, this is not always the case; for example, the *CYP2D6* inhibitor, fluoxetine, increased risperidone exposure in *CYP2D6* PMs as well EMs, possibly due to the effects of its metabolite (norfluoxetine) on a secondary pathway of risperidone metabolism (CYP3A4) [48, 49].

Moving forward, it will be important to characterise DDGIs and understand their clinical sequelae in larger patient datasets. In this study, despite a univariate signal, no association between substantial interactions and MACE was observed. Thus, larger patient cohorts and additional use of other endpoints potentially more directly related to interactions, such as (causality-assessed) adverse drug reactions and all-cause hospitalisation, should be utilised. Irrespective, as pharmacogenomics becomes more available in clinical practice, prescribers will be faced with combining genetic, co-medication, and comorbidity (e.g. renal/hepatic impairment) information together when making prescribing decisions. This is not trivial, and prescribers should ideally be supported with electronic clinical decision support systems, which in turn will often require increasing interoperability between different healthcare information systems (e.g. between primary/secondary care, and blood test results/medication data) alongside national and international structures to review and implement evolving evidence. Increasing the clinical evidence base of specific DDGIs will help prioritise DDGIs for incremental implementation.

This study assessed multimorbidity, polypharmacy, and drug- and gene-based interactions within a UK-based NSTE-ACS cohort. However, it does have limitations. Beyond the limited patient sample size, mental illness diagnoses could not be included as comorbidities, no clinical frailty scale was recorded, and non-cardiovascular drug data were only established in a subset of patients.

## Conclusions

This study demonstrated that multimorbidity, polypharmacy, and drug interactions are common after a NSTE-ACS, with CYP2C19 and SLCO1B1 being involved in the largest number of identified interactions. Multimorbidity and drug use were linked to interactions and clinical events. Differences in prescribing patterns between patients based on age and gender were identified. The high prevalence of DDGIs emphasises the need for further DDGI research and for healthcare systems to consider integration of medication information with genetic data and other clinical factors to enhance medicines optimisation.

## Supplementary Information

**Additional file 1.** Table of drug-metabolising CYP substrates.

**Additional file 2.** Table of drug-metabolising CYP inhibitors.

**Additional file 3.** Table of drug-metabolising CYP inducers.

**Additional file 4.** Table of transporter substrates, inhibitors and inducers.

**Additional file 5.** Table of genotypes considered and actionable genotype-based metaboliser phenotypes for each drug.

**Additional file 6.** Table of clinical characteristics, multimorbidity and medication use by age.

**Additional file 7.** Visualisation of non-linear relationships with age.

**Additional file 8.** Table of all identified interactions.

**Additional file 9.** Table of identified substantial interactions.

**Additional file 10.** Table of associations with patients that have at least one identified drug interaction.

**Additional file 11.** Sensitivity analysis after substitution of dichotomous multimorbidity (≥2 comorbidities) with number of comorbidities.

## Data Availability

The datasets used and analysed during the current study are available from the corresponding author on reasonable request.

## Abbreviations

ACEI: Angiotensin-converting enzyme inhibitor
ACM: All-cause mortality
ARB: Angiotensin II receptor blocker
AUC: Area under the concentration-time curve
BMI: Body mass index
CABG: Coronary artery bypass graft surgery
CHD: Coronary heart disease
CI: Confidence interval
CNV: Copy number variant
CPIC: Clinical Pharmacogenetics Implementation Consortium
CYP: Cytochrome P450 enzyme
DDI: Drug-drug interaction
DDGI: Drug-drug-gene interaction
DGI: Drug-gene interaction
DGGI: Drug-gene-gene interaction
DPWG: Dutch Pharmacogenetics Working Group
EM: Extensive metaboliser
FDA: US Food and Drug Administration
HET: Heterozygous
HOM: Homozygous for the variant allele
HR: Hazard ratio
IM: Intermediate metaboliser
IQR: Interquartile range
MACE: Major adverse cardiovascular events
MI: Myocardial infarction
NSTE-ACS: Non-ST elevation acute coronary syndrome
PCI: Percutaneous coronary intervention
PhACS: Pharmacogenetics of Acute Coronary Syndrome study
PM: Poor metaboliser
QC: Quality control
RM: Rapid metaboliser
UM: Ultra-rapid metaboliser
