# Supplementary material for: Multimorbidity, polypharmacy, and drug-drug-gene interactions following a non-ST elevation acute coronary syndrome: analysis of a multicentre observational study
Source: BMC Med. 2020 Nov 25;18:367. doi: 10.1186/s12916-020-01827-z (PMC7687685; doi:10.1186/s12916-020-01827-z)
Supplement: Supplementary file 1 — Additional file 1. Table of drug-metabolising CYP substrates. [file 12916_2020_1827_MOESM1_ESM.docx]

**Additional file 1. Table of drug-metabolising CYP substrates**

| **CYP1A2** | **CYP3A4/5** | **CYP2B6** | **CYP2C8** | **CYP2C9** | **CYP2C19** | **CYP2D6** |
| --- | --- | --- | --- | --- | --- | --- |
| **Sensitive substrates**† | | | | | | |
| Alosetron | Alfentanil | Bupropion | Repaglinide | Celecoxib | Omeprazole | Atomoxetine |
| Caffeine | Avanafil |  |  |  | Mephenytoin | Desipramine |
| Duloxetine | Budesonide |  |  |  |  | Dextromethorphan |
| Melatonin | Buspirone |  |  |  |  | Eliglustat |
| Ramelteon | Conivaptan |  |  |  |  | Nibivolol |
| Tasimelteon | Darifenacin |  |  |  |  | Nortriptyline |
| Theophylline | Darunavir |  |  |  |  | Perphenazine |
| Tizanidine | Dasatinib |  |  |  |  | Tolterodine |
|  | Dronedarone |  |  |  |  | Venlafaxine |
|  | Ebastine |  |  |  |  |  |
|  | Eletriptan |  |  |  |  |  |
|  | Eplerenone |  |  |  |  |  |
|  | Everolimus |  |  |  |  |  |
|  | Felodipine |  |  |  |  |  |
|  | Ibrutinib |  |  |  |  |  |
|  | Indinavir |  |  |  |  |  |
|  | Lomitapide |  |  |  |  |  |
|  | Lovastatin |  |  |  |  |  |
|  | Lurasidone |  |  |  |  |  |
|  | Maraviroc |  |  |  |  |  |
|  | Midazolam |  |  |  |  |  |
|  | Naloxegol |  |  |  |  |  |
|  | Nisoldipine |  |  |  |  |  |
|  | Quetiapine |  |  |  |  |  |
|  | Saquinavir |  |  |  |  |  |
|  | Sildenafil |  |  |  |  |  |
|  | Simvastatin |  |  |  |  |  |
|  | Sirolimus |  |  |  |  |  |
|  | Tacrolimus |  |  |  |  |  |
|  | Ticagrelor |  |  |  |  |  |
|  | Tipranavir |  |  |  |  |  |
|  | Tolvaptan |  |  |  |  |  |
|  | Triazolam |  |  |  |  |  |
|  | Vardenafil |  |  |  |  |  |
| **Moderate sensitive substrates**†† | | | | | | |
| **CYP1A2** | **CYP3A4/5** | **CYP2B6** | **CYP2C8** | **CYP2C9** | **CYP2C19** | **CYP2D6** |
| Clozapine | Alprazolam | Efavirenz | Montelukast | Glimepiride | Diazepam | Amitriptyline |
| Pirfenidone | Aprepitant |  | Pioglitazone | Phenytoin | Lansoprazole | Encainide |
| Ramosetron | Atorvastatin |  | Rosiglitazone | Tolbutamide | Rabeprazole | Imipramine |
|  | Colchicine |  |  | Warfarin | Voriconazole | Metoprolol |
|  | Eliglustat |  |  |  |  | Propafenone |
|  | Pimozide |  |  |  |  | Propranolol |
|  | Rilpivirine |  |  |  |  | Tramadol |
|  | Rivaroxaban |  |  |  |  | Trimipramine |
|  | Tadalafil |  |  |  |  |  |
| **Other substrates**††† | | | | | | |
| **CYP1A2** | **CYP3A4/5** | **CYP2B6** | **CYP2C8** | **CYP2C9** | **CYP2C19** | **CYP2D6** |
| Cyclobenzaprine | Astemizole | Artemisinin | Amodiaquine | Diclofenac | Amitriptyline | Aripiprazole |
| Fluvoxamine | Chlorphenamine | Cyclophosphamide | Paclitaxel | Fluvastatin | Carisoprodol | Carvedilol |
| Haloperidol | Ciclosporin | Ifosfamide | Torsemide | Glibenclamide | Citalopram | Clomipramine |
| Imipramine | Cisapride | Ketamine |  | Glipizide | Clomipramine | Codeine |
| Mexiletine | Clarithromycin | Meperidine |  | Ibuprofen | Clopidogrel | Despiramine |
| Nabumetone | Diazepam | Methadone |  | Irbesartan | Cyclophosphamide | Doxepine |
| Naproxen | Erythromycin | Nevirapine |  | Losartan | Esomeprazole | Duloxetine |
| Olanzapine | Nevirapine | Propofol |  | Naproxen | Imipramine | Flecainide |
| Riluzole | Quinidine | Selegiline |  | Piroxicam | Labetalol | Fluoxetine |
| Tacrine | Ritonavir |  |  | Rosiglitazone | Pantoprazole | Haloperidol |
| Triamterene | Telithromycin |  |  | Torsemide | Phenobarbital | Mexiletine |
| Zileuton |  |  |  | Valproic acid | Phenytoin | Ondansetron |
| Zolmitriptan |  |  |  | Zafirlukast | Proguanil | Oxycodone |
|  |  |  |  |  |  | Paroxetine |
|  |  |  |  |  |  | Risperidone |
|  |  |  |  |  |  | Tamoxifen |
|  |  |  |  |  |  | Thioridazine |
|  |  |  |  |  |  | Timolol |

† = Sensitive substrates are taken from the FDA clinical substrates table [15] and experience an increase in area under the concentration-time curve (AUC) of ≥5-fold with strong index inhibitors of a given metabolic pathway.

†† = Moderate sensitive substrates are taken from the FDA clinical substrates table [15] and experience an increase in AUC of ≥2 to <5-fold with strong index inhibitors of a given metabolic pathway.

††† = The substrates listed in the ‘Other’ section are drugs taken from the Indiana Flockhart Table^TM^ [16] not present in the FDA clinical substrates table [15].
