# Supplementary material for: Multimorbidity, polypharmacy, and drug-drug-gene interactions following a non-ST elevation acute coronary syndrome: analysis of a multicentre observational study
Source: BMC Med. 2020 Nov 25;18:367. doi: 10.1186/s12916-020-01827-z (PMC7687685; doi:10.1186/s12916-020-01827-z)
Supplement: Supplementary file 2 — Additional file 2. Table of drug-metabolising CYP inhibitors. [file 12916_2020_1827_MOESM2_ESM.docx]

**Additional file 2. Table of drug-metabolising CYP inhibitors**

| **CYP1A2** | **CYP3A4/5** | **CYP2B6** | **CYP2C8** | **CYP2C9** | **CYP2C19** | **CYP2D6** |
| --- | --- | --- | --- | --- | --- | --- |
| **Strong inhibitors**† | | | | | | |
| Ciprofloxacin | Boceprevir | / | Clopidogrel | / | Esomeprazole | Bupropion |
| Enoxacin | Clarithromycin |  | Gemfibrozil |  | Fluconazole | Fluoxetine |
| Fluvoxamine | Cobicistat |  |  |  | Fluoxetine | Paroxetine |
| Zafirlukast | Conivaptan |  |  |  | Fluvoxamine | Quinidine |
|  | Diltiazem |  |  |  | Omeprazole | Terbinafine |
|  | Grapefruit juice |  |  |  | Ticlopidine |  |
|  | Idelalisib |  |  |  |  |  |
|  | Indinavir |  |  |  |  |  |
|  | Itraconazole |  |  |  |  |  |
|  | Ketoconazole |  |  |  |  |  |
|  | Nefazodone |  |  |  |  |  |
|  | Nelfinavir |  |  |  |  |  |
|  | Posaconazole |  |  |  |  |  |
|  | Ritonavir |  |  |  |  |  |
|  | Troleandomycin |  |  |  |  |  |
|  | Voriconazole |  |  |  |  |  |
| **Moderate inhibitors**†† | | | | | | |
| **CYP1A2** | **CYP3A4/5** | **CYP2B6** | **CYP2C8** | **CYP2C9** | **CYP2C19** | **CYP2D6** |
| Methoxsalen | Aprepitant | / | Deferasirox | Amiodarone | / | Cinacalcet |
| Mexiletine | Cimetidine |  | Teriflunomide | Felbamate |  | Duloxetine |
| Oral contraceptives | Ciprofloxacin |  |  | Fluconazole |  | Fluvoxamine |
|  | Clotrimazole |  |  | Miconazole |  | Mirabegron |
|  | Crizotinib |  |  | Piperine |  |  |
|  | Ciclosporin |  |  |  |  |  |
|  | Dronedarone |  |  |  |  |  |
|  | Erythromycin |  |  |  |  |  |
|  | Fluconazole |  |  |  |  |  |
|  | Fluvoxamine |  |  |  |  |  |
|  | Imatinib |  |  |  |  |  |
|  | Tofisopam |  |  |  |  |  |
|  | Verapamil |  |  |  |  |  |
| **Weak inhibitors**††† | | | | | | |
| **CYP1A2** | **CYP3A4/5** | **CYP2B6** | **CYP2C8** | **CYP2C9** | **CYP2C19** | **CYP2D6** |
| Acyclovir | Chlorzoxazone | Clopidogrel | Telithromycin | Diosmin | Voriconazole | Abiraterone |
| Allopurinol | Cilostazol | Tenofovir | Trimethoprim | Disulfiram |  | Amiodarone |
| Cimetidine | Fosaprepitant | Ticlopidine |  | Fluvastatin |  | Celecoxib |
| Peginterferon 2a | Istradefylline | Voriconazole |  | Fluvoxamine |  | Cimetidine |
| Piperine | Ivacaftor |  |  | Voriconazole |  | Clobazam |
| Zileuton | Lomitapide |  |  |  |  | Cobicistat |
|  | Ranitidine |  |  |  |  | Desvenlafaxine |
|  | Ranolazine |  |  |  |  | Escitalopram |
|  | Tacrolimus |  |  |  |  | Labetalol |
|  | Ticagrelor |  |  |  |  | Lorcaserin |
|  |  |  |  |  |  | Ritonavir |
|  |  |  |  |  |  | Sertraline |
|  |  |  |  |  |  | Vemurafenib |
| **Other inhibitors**‡ | | | | | | |
| **CYP1A2** | **CYP3A4/5** | **CYP2B6** | **CYP2C8** | **CYP2C9** | **CYP2C19** | **CYP2D6** |
| Amiodarone | Amiodarone | Thiotepa | Montelukast | Efavirenz | Cimetidine | Aripiprazole |
| Efavirenz | Suboxone |  |  | Isoniazid | Felbamate | Chlorphenamine |
| Ticlopidine |  |  |  | Metronidazole | Isoniazid | Clomipramine |
| Levofloxacin |  |  |  | Paroxetine | Ketoconazole | Diphenhydramine |
|  |  |  |  | Sulfamethoxazole | Lansoprazole | Doxepin |
|  |  |  |  |  | Oral contraceptives | Haloperidol |
|  |  |  |  |  | Pantoprazole | Methadone |

Strong (†), moderate (††) and weak (†††) inhibitors increase the area under the concentration-time curve (AUC) of sensitive index substrates of a given metabolic pathway by ≥5-fold, ≥2 to <5-fold, and ≥1.25 to <2-fold, respectively.

The strong, moderate, and weak inhibitors were taken almost exclusively from the FDA Tables [15], although esomeprazole and omeprazole were designated as strong CYP2C19 inhibitors based on the Flockhart Table^TM^ [16] and Modak *et* al,2016 [27].

‡ **=** The inhibitors listed in the ‘Other’ section are drugs taken from the Indiana Flockhart Table^TM^ [16] that are not present in the FDA Tables [15] and whose inhibitory strength has not been confirmed.
