# Supplementary material for: Multimorbidity, polypharmacy, and drug-drug-gene interactions following a non-ST elevation acute coronary syndrome: analysis of a multicentre observational study
Source: BMC Med. 2020 Nov 25;18:367. doi: 10.1186/s12916-020-01827-z (PMC7687685; doi:10.1186/s12916-020-01827-z)
Supplement: Supplementary file 3 — Additional file 3. Table of drug-metabolising CYP inducers. [file 12916_2020_1827_MOESM3_ESM.docx]

**Additional file 3. Table of drug-metabolising CYP inducers**

| **CYP1A2** | **CYP3A4/5** | **CYP2B6** | **CYP2C8** | **CYP2C9** | **CYP2C19** | **CYP2D6** |
| --- | --- | --- | --- | --- | --- | --- |
| **Strong inducers**† | | | | | | |
| / | Carbamazepine | Carbamazepine | / | / | Rifampicin | / |
|  | Enzalutamide |  |  |  | Ritonavir |  |
|  | Mitotane |  |  |  |  |  |
|  | Phenytoin |  |  |  |  |  |
|  | Rifampicin |  |  |  |  |  |
|  | St. John's Wort |  |  |  |  |  |
| **Moderate inducers**†† | | | | | | |
| Phenytoin | Bosentan | Efavirenz | Rifampicin | Aprepitant | Efavirenz | / |
| Rifampicin | Efavirenz | Rifampicin |  | Carbamazepine | Enzalutamide |  |
| Ritonavir | Etravirine | Ritonavir |  | Enzalutamide | Phenytoin |  |
| Tobacco | Modafinil |  |  | Rifampicin |  |  |
| Teriflunomide |  |  |  | Ritonavir |  |  |
| **Weak inducers**††† | | | | | | |
| / | Armodafinil | Nevirapine | / | / | / | / |
|  | Rufinamide |  |  |  |  |  |
| **Other inducers**‡ | | | | | | |
| Carbamazepine | Nevirapine | Artemisinin | / | Nevirapine | St. John's Wort | / |
|  | Phenobarbital | Phenobarbital |  | Phenobarbital |  |  |
|  | Pioglitazone | Phenytoin |  | St. John's Wort |  |  |
|  | Rifabutin |  |  |  |  |  |
|  | Troglitazone |  |  |  |  |  |

Strong (†), moderate (††) and weak (†††) inducers are from the clinical inducers FDA table [15] and decrease the area under the concentration-time curve (AUC) of sensitive index substrates of a given metabolic pathway by ≥80%, ≥50% to <80%, and ≥20% to <50%, respectively.

‡ **=** The inducers listed in the ‘Other’ section are drugs taken from the Indiana Flockhart Table^TM^ [16] not present in the FDA Tables [15].
