# Supplementary material for: Multimorbidity, polypharmacy, and drug-drug-gene interactions following a non-ST elevation acute coronary syndrome: analysis of a multicentre observational study
Source: BMC Med. 2020 Nov 25;18:367. doi: 10.1186/s12916-020-01827-z (PMC7687685; doi:10.1186/s12916-020-01827-z)
Supplement: Supplementary file 4 — Additional file 4. Table of transporter substrates, inhibitors and inducers. [file 12916_2020_1827_MOESM4_ESM.docx]

**Additional file 4. Table of transporter substrates, inhibitors and inducers**

| **ABCB1 (P-gp)** | | **SLCO1B1** | |
| --- | --- | --- | --- |
| **Substrates** [15,28] | **Inhibitors** [15] | **Substrates** [15] | **Inhibitors** [15] |
| Actinomycin D | **Strong** | Asunaprevir | **Strong** |
| Aliskiren | Amiodarone | Atorvastatin | Atazanavir & ritonavir |
| Ambrisentan | Carvedilol | Bosentan | Ciclosporin |
| Apixaban | Dronedarone | Danoprevir | Lopinavir & ritonavir |
| Atorvastatin | Itraconazole | Docetaxel | Rifampicin |
| Bepridil | Lopinavir & ritonavir | Fexofenadine | **Others** |
| Berberine | Quinidine | Glibenclamide | Clarithromycin |
| Celiprolol | Ritonavir | Nateglinide | Erythromycin |
| Ciclosporin | Saquinavir | Paclitaxel | Gemfibrozil |
| Cimetidine | Verapamil | Pitavastatin | Simeprevir |
| Ciprofloxacin | **Others** | Pravastatin |  |
| Clopidogrel | Clarithromycin | Repaglinide | **Inducers** |
| Colchicine | Lapatinib | Rosuvastatin | **Strong** |
| Dabigatran | Propafenone | Simvastatin | / |
| Daunorubicin | Ranolazine |  | **Others** |
| Digoxin | Telaprevir |  | / |
| Diltiazem | Tipranavir |  |  |
| Domperidone |  |  |  |
| Doxorubicin | **Inducers** [28] |  |  |
| Edoxaban | **Strong** |  |  |
| Erythromycin | Carbamazepine |  |  |
| Etoposide | Dexamethasone |  |  |
| Everolimus | Doxorubicin |  |  |
| Fexofenadine | Phenytoin |  |  |
| Imatinib | Rifampicin |  |  |
| Irinotecan | St. John's Wort |  |  |
| Ivermectin | **Others** |  |  |
| Labetalol | Ciclosporin |  |  |
| Lapatinib | Tipranavir |  |  |
| Lidocaine | Venlafaxine |  |  |
| Loperamide |  |  |  |
| Losartan |  |  |  |
| Lovastatin |  |  |  |
| Maraviroc |  |  |  |
| Methotrexate |  |  |  |
| Mitomycin C |  |  |  |
| Nadolol |  |  |  |
| Nilotinib |  |  |  |
| Ondansetron |  |  |  |
| Paclitaxel |  |  |  |
| Posaconazole |  |  |  |
| Propranolol |  |  |  |
| Quinine Sulfate |  |  |  |
| Rifampicin |  |  |  |
| Rivaroxaban |  |  |  |
| Saxagliptin |  |  |  |
| Tacrolimus |  |  |  |
| Talinolol |  |  |  |
| Taxol |  |  |  |
| Terfenadine |  |  |  |
| Ticagrelor |  |  |  |
| Timolol |  |  |  |
| Tolvaptan |  |  |  |
| Topotecan |  |  |  |
| Verapamil |  |  |  |
| Vinblastine |  |  |  |
| Vincristine |  |  |  |
| Warfarin |  |  |  |

The assessment of strength of P-gp inhibitors was from Wessler *et al*, 2013 [28] and Appendix A of the British Columbia guidelines on Potential NOAC Drug Interactions [29]; the latter was also used to determine the strength of P-gp inducers [29]. Assessment of strength of SLCO1B1 (OATP1B1) inhibitors from Karlgren *et al*, 2012 [30].
