# Supplementary material for: Multimorbidity, polypharmacy, and drug-drug-gene interactions following a non-ST elevation acute coronary syndrome: analysis of a multicentre observational study
Source: BMC Med. 2020 Nov 25;18:367. doi: 10.1186/s12916-020-01827-z (PMC7687685; doi:10.1186/s12916-020-01827-z)
Supplement: Supplementary file 5 — Additional file 5. Table of genotypes considered and actionable genotype-based metaboliser phenotypes for each drug. [file 12916_2020_1827_MOESM5_ESM.docx]

**Additional file 5.** **Table of genotypes considered and actionable genotype-based metaboliser phenotypes for each drug**

| **Drug** | **Gene** | **Variant(s)** | **Actionable genotype-based phenotype(s)** | | | | | **Guideline(s) used** |
| --- | --- | --- | --- | --- | --- | --- | --- | --- |
|  |  |  | **PM** | **IM** | **EM** | **RM** | **UM** |  |
| Amitriptyline† | *CYP2D6* | *3, *4, *5, *9, *10, *41, xN | Yes | Yes | No | - | Yes | CPIC [31] & DPWG [32] |
|  | *CYP2C19* | *2, *17 | Yes | No | No | Yes | Yes | CPIC [31] |
| Aripiprazole | *CYP2D6* | *3, *4, *5, *9, *10, *41, xN | Yes | No | No | - | No | DPWG [32] |
| Atorvastatin† | *SLCO1B1* | rs4149056 (c.521T>C) | Yes | Yes | No | - | - | DPWG [32] |
| Azathioprine | *TPMT* | *3A, *3B, *3C | Yes | Yes | No | - | - | CPIC [33] |
| Citalopram† | *CYP2C19* | *2, *17 | Yes | No | No | Yes | Yes | CPIC [34] |
| Clomipramine† | *CYP2D6* | *3, *4, *5, *9, *10, *41, xN | Yes | Yes | No | - | Yes | CPIC [31] & DPWG [32] |
|  | *CYP2C19* | *2, *17 | Yes | No | No | Yes | Yes | CPIC [31] |
| Clopidogrel† | *CYP2C19* | *2, *17 | Yes | Yes | No | No | No | CPIC [35] |
| Codeine† | *CYP2D6* | *3, *4, *5, *9, *10, *41, xN | Yes | No | No | - | Yes | CPIC [36] & DPWG [32] |
| Escitalopram | *CYP2C19* | *2, *17 | Yes | No | No | Yes | Yes | CPIC [34] |
| Imipramine† | *CYP2D6* | *3, *4, *5, *9, *10, *41, xN | Yes | Yes | No | - | Yes | CPIC [31] & DPWG [32] |
|  | *CYP2C19* | *2, *17 | Yes | No | No | Yes | Yes | CPIC [31] |
| Lansoprazole† | *CYP2C19* | *2, *17 | No | No | No | Yes | Yes | DPWG [32] |
| Metoprolol† | *CYP2D6* | *3, *4, *5, *9, *10, *41, xN | Yes | Yes | No | - | Yes | DPWG [32] |
| Nortriptyline | *CYP2D6* | *3, *4, *5, *9, *10, *41, xN | Yes | Yes | No | - | Yes | CPIC [31] & DPWG [32] |
| Oestrogens | *F5* | rs6025 (c.1601G>A) | Yes | Yes | No | - | - | DPWG [32] |
| Omeprazole† | *CYP2C19* | *2, *17 | No | No | No | Yes | Yes | DPWG [32] |
| Pantoprazole† | *CYP2C19* | *2, *17 | No | No | No | Yes | Yes | DPWG [32] |
| Paroxetine | *CYP2D6* | *3, *4, *5, *9, *10, *41, xN | Yes | No | No | - | Yes | CPIC [34] |
| Phenytoin† | *CYP2C9* | *2, *3 | Yes | Yes | No | - | - | CPIC [37] |
| Sertraline† | *CYP2C19* | *2, *17 | Yes | No | No | Yes | Yes | CPIC [34] |
| Simvastatin† | *SLCO1B1* | rs4149056 (c.521T>C) | Yes | Yes | No | - | - | CPIC [38] & DPWG [32] |
| Tacrolimus† | *CYP3A5* | *3 | No | Yes | Yes | - | - | CPIC [39] |
| Tamoxifen† | *CYP2D6* | *3, *4, *5, *9, *10, *41, xN | Yes | Yes | No | - | No | CPIC [40] & DPWG [32] |
| Tramadol | *CYP2D6* | *3, *4, *5, *9, *10, *41, xN | No | No | No | - | Yes | DPWG [32] |
| Warfarin† | *VKORC1* | rs9934438 (c.1173C>T) | Yes | Yes^a^ | No | - | - | Warfarin product label [41] |
|  | CYP2C9 | *2, *3 | Yes | Yes^b^ | No | - | - |  |

This table lists all drugs that have a pharmacogenomic clinical guideline from CPIC and/or DPWG for the included pharmacogenes [11], and one or more patient within the interaction cohort was recorded as taking the drug.

† = A drug for which at least one drug-gene interaction was identified within the interaction cohort.

CPIC = The Clinical Pharmacogenetics Implementation Consortium; DPWG = The Dutch Pharmacogenetics Working Group; EM = extensive (normal) metaboliser; IM = intermediate metaboliser; PM = poor metaboliser; RM = rapid metaboliser; UM = ultra-rapid metaboliser.

For non-CYP genes in this table: ‘IM’= carrier of one variant, and; ‘PM’ = homozygous/compound heterozygous for two variant alleles.

- = genotype-predicted (metaboliser) phenotype not recognised.

^a^ = Heterozygosity for *VKORC1* rs9934438 (genotype CT) is considered actionable only in those that also carry *CYP2C9*2* or *CYP2C9*3*; ^b^ = *CYP2C9 *1/*3* IM is actionable, but *CYP2C9 *1/*2* is actionable only in those that also carry the *VKORC1* rs9934438 variant allele;
