# Supplementary material for: Multimorbidity, polypharmacy, and drug-drug-gene interactions following a non-ST elevation acute coronary syndrome: analysis of a multicentre observational study
Source: BMC Med. 2020 Nov 25;18:367. doi: 10.1186/s12916-020-01827-z (PMC7687685; doi:10.1186/s12916-020-01827-z)
Supplement: Supplementary file 6 — Additional file 6. Table of clinical characteristics, multimorbidity and medication use by age. [file 12916_2020_1827_MOESM6_ESM.docx]

**Additional file 6. Table of clinical characteristics, multimorbidity and medication use by age**

| **Characteristic** | **<65 years old** | **≥65 years old** | **p-value** †† | **Age** ††† | | **Age squared** ††† | |
| --- | --- | --- | --- | --- | --- | --- | --- |
|  | **n=690** † | **n=760** † |  | **Risk estimate (95% CI)** | **p-value** | **Risk estimate (95% CI)** | **p-value** |
| **Demographics** | | | | | | | |
| Sex (male), n (%) | 533 (77.2) | 521 (68.6) | 1.7x10^-4^ | OR 1.03 (1.02-1.04) | 2.0x10^-8^ | - | - |
| BMI, median (IQR) | 28.4 (25.6-32.2) | 27.7 (24.5-31.1) | 8.1x10^-5^ | B -0.091 (-0.12 - -0.062) | 1.6x10^-9^ | - | - |
| **Comorbidities** | | | | | | | |
| Smoking, n (%) | 266 (38.6) | 99 (13.0) | 4.8x10^-27^ | OR 0.93 (0.92-0.94) | 6.0x10^-35^ | - | - |
| Cardiovascular multimorbidity, n (%) | 126 (18.2) | 266 (35.0) | 1.1x10^-12^ | OR 1.04 (1.03-1.05) | 2.1x10^-14^ | - | - |
| Non-cardiovascular multimorbidity, n (%) | 84 (12.2) | 181 (23.8) | 6.2x10^-8^ | OR 1.04 (1.03-1.05) | 4.7x10^-10^ | - | - |
| All multimorbidity, n (%) | 259 (37.5) | 466 (61.3) | 1.2x10^-18^ | OR 1.05 (1.04-1.06) | 1.8x10^-22^ | - | ‡ |
| Number of comorbidities, n (%) | | | | | | | |
| 0 | 225 (32.6) | 95 (12.5) | <2.0x10^-16^ | RR 1.02 (1.02-1.03) | <2.0x10^-16^ | RR 1.00 (1.00-1.00) | 1.2x10^-3^ |
| 1 | 206 (29.9) | 199 (26.2) |  |  |  |  |  |
| 2 | 133 (19.3) | 191 (25.1) |  |  |  |  |  |
| 3 | 78 (11.3) | 134 (17.6) |  |  |  |  |  |
| 4-6 | 45 (6.5) | 127 (16.7) |  |  |  |  |  |
| 7-9 | 3 (0.4) | 14 (1.8) |  |  |  |  |  |
| **Index NSTE-ACS** | | | | | | | |
| Raised troponin, n (%) | 656 (95.1) | 727 (95.7) | 0.91 | OR 1.00 (0.98-1.02) | 0.84 | - | - |
| Coronary catheterisation, n (%) | 509 (73.8) | 462 (60.8) | 3.1x10^-7^ | OR 0.97 (0.96-0.98) | 3.5x10^-12^ | OR 0.998 (0.998-0.999) | 7.0x10^-6^ |
| PCI/CABG, n (%) | 360 (52.2) | 296 (38.9) | 1.0x10^-6^ | OR 0.97 (0.96-0.98) | 2.2x10^-10^ | OR 0.998 (0.998-0.999) | 7.0x10^-6^ |
| **Cardiovascular drugs at discharge** | | | | | | | |
| Aspirin, n (%) | 667 (96.7) | 698 (91.8) | 3.1x10^-4^ | OR 0.95 (0.93-0.97) | 5.0x10^-6^ | - | - |
| P2Y_12_ inhibitor, n (%) | 612 (88.7) | 620 (81.6) | 5.4x10^-4^ | OR 0.98 (0.97-0.99) | 1.6x10^-3^ | - | - |
| ACEI/ARB, n (%) | 579 (83.9) | 607 (79.9) | 0.11 | OR 0.99 (0.98-1.00) | 0.093 | - | - |
| Beta blocker, n (%) | 589 (85.4) | 598 (78.7) | 8.4 x10^-3^ | OR 0.98 (0.97-0.99) | 1.5x10^-3^ | - | - |
| Statin, n (%) | 660 (95.7) | 711 (93.6) | 0.097 | OR 0.98 (0.96-0.996) | 0.019 | - | - |
| Patients on all five secondary prevention cardiovascular drugs, n (%) ‡ | 447 (64.8) | 386 (50.8) | 5.3x10^-7^ | OR 0.97 (0.96-0.98) | 1.5x10^-8^ | - | - |
| **Drug use by category at discharge** | | | | | | | |
| **N=698** ‡‡ | **n=318** | **n=380** |  |  | | | |
| Gastro-intestinal, n (%) | 164 (51.6) | 232 (61.1) | 0.018 | OR 1.03 (1.01-1.04) | 1.2x10^-4^ | - | - |
| All Cardiovascular, n (%) | 318 (100.0) | 379 (99.7) | 0.99 | OR 0.99 (0.83-1.17) | 0.89 | - | - |
| Respiratory, n (%) | 60 (18.9) | 84 (22.1) | 0.31 | OR 1.02 (1.00-1.03) | 0.075 | - | - |
| Central nervous system, n (%) | 118 (37.1) | 127 (33.4) | 0.23 | OR 0.99 (0.98-1.01) | 0.20 | - | - |
| Infections, n (%) | 19 (6.0) | 31 (8.2) | 0.29 | OR 1.02 (1.00-1.05) | 0.12 | - | - |
| Endocrine, n (%) | 69 (21.7) | 150 (39.5) | 2.0x10^-6^ | OR 1.04 (1.02-1.05) | 4.1x10^-7^ | - | - |
| Nutrition & blood, n (%) | 30 (9.4) | 77 (20.3) | 2.3x10^-4^ | OR 1.04 (1.02-1.06) | 2.0x10^-4^ | - | - |
| Musculoskeletal, n (%) | 34 (10.7) | 67 (17.6) | 0.014 | OR 1.04 (1.02-1.06) | 2.5x10^-4^ | - | - |
| Eye, ear, nose, oropharynx & skin, n (%) | 11 (3.5) | 27 (7.1) | 0.034 | OR 1.06 (1.03-1.10) | 1.2x10^-4^ | - | - |
| Other, n (%) | 6 (1.9) | 44 (11.6) | 8.0x10^-6^ | OR 1.06 (1.04-1.09) | 9.0x10^-6^ | - | - |
| Number of cardiovascular drugs/patient, median (IQR, range) | 6 (5-6, 1-9) | 6 (5-7, 0-9) | 1.1x10^-4^ | RR 1.00 (1.00-1.00) | 1.8x10^-5^ | - | - |
| Number of non-cardiovascular drugs/patient, median (IQR, range) | 2 (1-4, 0-11) | 2 (1-5, 0-16) | 7.1x10^-5^ | RR 1.02 (1.01-1.02) | 1.9x10^-7^ | - | - |
| Number of drugs/patient, median (IQR, range) | 8 (7-10, 4-18) | 9 (8-12, 2-26) | 3.0x10^-6^ | RR 1.01 (1.00-1.01) | 2.1x10^-8^ | - | - |

ACEI = angiotensin-converting enzyme inhibitor; ARB = angiotensin II receptor blocker; B = unstandardised coefficient; CABG = coronary artery bypass graft surgery; IQR = interquartile range; n (%) = number (per cent) of patients; OR = odds ratio; PCI = percutaneous coronary intervention; RR= relative risk.

† = The age of six patients was missing from the n=1456 cohort; thus, these patients were not included in the age-stratified columns.

†† = p-values to describe the association between dichotomised age (≥65 vs <65 years) and each variable, adjusted for patient sex.

††† = Associations between age (as a continuous variable) and each dependent variable, adjusted for patient sex. Non-linearity in the association between age and each dependent variable was investigated. If non-linearity was identified, mean centred age and mean centred age squared, adjusted for sex, are reported. Identification of non-linearity involved visual inspection and mean centred age squared p<0.05. If non-linearity was not detected, age (not mean centred) adjusted for patient sex is reported.

To aid interpretation, an odds ratio (or relative risk) <1 for age squared is derived from a negative coefficient and thus indicates a decline (e.g. a decrease after an increase, or increase in the rate of decrease) in the dependent variable (e.g. PCI/CABG) after a certain age.

‡ = for all multimorbidity, a weak non-linear association with age was suggested visually, although age squared was only borderline statistically significant (p=0.065), and so the non-linear relationship was omitted.

‡ = Secondary prevention cardiovascular drugs were: aspirin, P2Y_12_ inhibitor, ACEI/ARB, beta blocker, and a statin.

‡‡ = For drug categories within the n=698 cohort, a patient was counted if they were on one or more drugs within a given category.
