# Supplementary figures and images for: Multimorbidity, polypharmacy, and drug-drug-gene interactions following a non-ST elevation acute coronary syndrome: analysis of a multicentre observational study
Source: BMC Med. 2020 Nov 25;18:367. doi: 10.1186/s12916-020-01827-z (PMC7687685; doi:10.1186/s12916-020-01827-z)

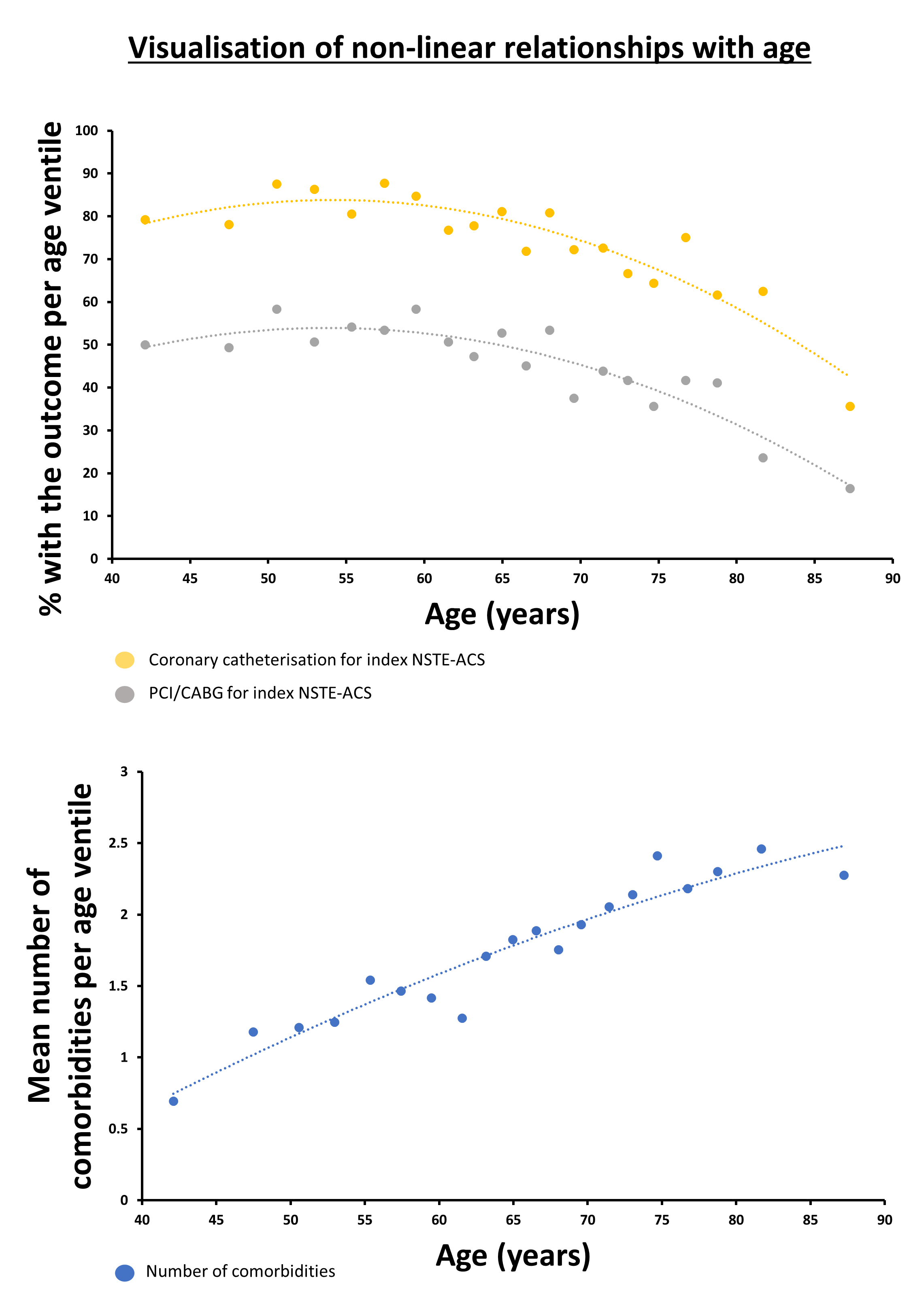

Supplement: Supplementary file 7 — Additional file 7. Visualisation of non-linear relationships with age. [file 12916_2020_1827_MOESM7_ESM.tif]
