# Supplementary material for: Multimorbidity, polypharmacy, and drug-drug-gene interactions following a non-ST elevation acute coronary syndrome: analysis of a multicentre observational study
Source: BMC Med. 2020 Nov 25;18:367. doi: 10.1186/s12916-020-01827-z (PMC7687685; doi:10.1186/s12916-020-01827-z)
Supplement: Supplementary file 8 — Additional file 8. Table of all identified interactions. [file 12916_2020_1827_MOESM8_ESM.docx]

**Additional file 8. Table of all identified interactions**

|  | **Protein level interaction** | | | | | | | | | **Total at drug level** |
| --- | --- | --- | --- | --- | --- | --- | --- | --- | --- | --- |
|  | **CYP1A2** | **CYP2C8** | **CYP2C9** | **CYP2C19** | **CYP2D6** | **CYP3A** | **P-gp** | **SLCO1B1** | **VKORC1** |  |
| **i) Number of interactions** | | | | | | | | | | |
| **Substrates** | 9 | 13 | 98 | 894 | 128 | 651 | 1240 | 614 | 32 | **1840** |
| **Inhibitors** | 60 | 560 | 31 | 300 | 64 | 127 | 49 | 7 | - | **1059** |
| **Inducers** | 192 | 0 | 6 | 2 | - | 15 | 15 | 0 | - | **23** |
| **Autoinhibitors** | 0 | 3 | 0 | 288 | 20 | 8 | 6 | - | - | **325** |
| **Autoinducers** | - | - | - | 2 | - | 0 | 1 | - | - | **3** |
| **Every inhibitory DDI^a^** | 1 | 13 | 5 | 273 | 11 | 124 | 82 | 7 | - | **474** |
| **Every induction DDI^a^** | 3 | 0 | 1 | 2 | - | 13 | 30 | 0 | - | **42** |
| **Total every DDI^a^** | 4 | 13 | 6 | 275 | 11 | 135 | 112 | 7 | - | **514** |
| **Total simple DDI^b^** | 4 | 13 | 6 | 202 | 10 | 105 | 91 | 6 | - | **397** |
| **Every reduction-of-function DGI^a^** | - | - | 8 | 163 | 17 | - | - | 175 | 7 | **370** |
| **Every gain-of-function DGI^a^** | - | - | - | 122 | 2 | 1 | - | - |  | **125** |
| **Total every DGI^a^** | - | - | 8 | 285 | 19 | 1 | - | 175 | 7 | **495** |
| **Total simple DGI^b^** | - | - | 3 | 205 | 10 | 1 | - | 141 | 3 | **363** |
| **DDGI: inhibition of 1 gene product** | - | - | 0 | 56 | 1 | 0 | - | 0 | - | **57** |
| **DDGI: opposing effect on 1 gene product** | - | - | 0 | 10 | 0 | 0 | - | - | - | **10** |
| **DDGI: inhibition of ≥2 gene products** | - | - | 1 | 3 | 2 | 0 | - | 28 | - | **34** |
| **DDGI: opposing effects on 2 gene products** | - | - | 0 | 5 | 0 | 0 | - | 6 | - | **11** |
| **Total DDGI** | - | - | 1 | 74 | 3 | 0 | - | 34 | - | **112** |
| **DGGIs** | - | - | 4 | 6 | 6 | 0 | - | 0 | 4 | **10** |
| **Overall Total (DDI or DGI or DDGI or DGGI)^c^** | **4** | **13** | **14** | **487** | **29** | **106** | **91** | **181** | **7** | **882** |
| **ii) Number of patients with at least one interaction per gene** | | | | | | | | | | |
| **Every DDI^a^** | 4 | 13 | 5 | 243 | 9 | 115 | 58 | 7 | - | **342** |
| **Simple DDI^b^** | 4 | 13 | 5 | 184 | 8 | 90 | 56 | 6 |  | **284** |
| **Every DGI^a^** | - | - | 8 | 275 | 19 | 1 | - | 175 | 7 | **384** |
| **Simple DGI^b^** | - | - | 3 | 204 | 10 | 1 | - | 141 | 3 | **302** |
| **DDGI** | - | - | 1 | 74 | 3 | 0 | - | 34 | - | **106** |
| **DGGI** | - | - | 4 | 6 | 6 | 0 | - | 0 | 4 | **10** |
| **Total^d^** | **4** | **13** | **13** | **369** | **27** | **115** | **58** | **181** | **7** | **503** |

This table shows, i) all interactions detected, and; ii) the number of patients with at least one interaction mediated per enzyme/transporter, from 652 post-NSTE-ACS patients.

^a^ = ‘Every’ DDI and DGI rows incorporate each identified DDI/DGI without exception, including those that are constituents of DDGIs/DGGIs.

^b^ = ‘Simple’ DDI and DGI rows exclude DDIs/DGIs that are constituents of DDGIs/DGGIs, and so only include DDIs or DGIs not considered in more complex interactions.

^c^ = the ‘Overall Total’ adds the different types of interaction together. Within a given enzyme/transporter column, an interaction is only counted once (e.g. a DDGI is counted as one interaction in this row, rather than three [a DDGI, DDI and DGI]). The final column on the table right-hand side provides the total number of interactions at the ‘drug-level’ (see below).

^d^ = gives the total number of patients with at least one type of interaction per enzyme/transporter, and at the drug-level (final column – see below).

Autoinhibition/autoinduction was not considered an interaction and so were not included in the DDI counts. DDGIs due to an opposing effect on one gene product were due to concomitant administration of a CYP2C19 inhibitor in *CYP2C19* rapid or ultra-rapid metaboliser patients. DDGIs mediated through two different enzymes/transporters were assigned to the gene involved in the component DGI. For atorvastatin (*SLCO1B1*) and clopidogrel (*CYP2C19*), a few DDDGIs were identified, which were included within the relevant DDGI row. Each DGGI was reported in the column of both genes involved (e.g. *CYP2C9* and *VKORC1* for warfarin), but was only counted as one interaction. The CYP1A2 induction DDIs were attributable to tobacco smoking. CYP2B6 was omitted from the table as no patient was on a CYP2B6 substrate.

Although no genomic variants within *ABCB1* (P-gp) or *CYP3A* (except *CYP3A5*3*-tacrolimus) were considered, several DDIs mediated via P-gp or CYP3A were nevertheless part of DDGIs attributed to other enzymes/transporters (e.g. CYP2C19 or SLCO1B1).

As two drugs often interact with one another through more than one enzyme/transporter (e.g. verapamil inhibits both CYP3A metabolism and P-gp efflux of atorvastatin), the last column on the right-hand side provides total numbers of interactions/number of patients with at least one interaction at the ‘drug level’ rather than at the level of each enzyme/transporter. - = category not applicable.
