# Supplementary material for: Multimorbidity, polypharmacy, and drug-drug-gene interactions following a non-ST elevation acute coronary syndrome: analysis of a multicentre observational study
Source: BMC Med. 2020 Nov 25;18:367. doi: 10.1186/s12916-020-01827-z (PMC7687685; doi:10.1186/s12916-020-01827-z)
Supplement: Supplementary file 9 — Additional file 9. Table of identified substantial interactions. [file 12916_2020_1827_MOESM9_ESM.docx]

**Additional file 9. Table of identified substantial interactions**

|  | **Protein level interaction** | | | | | | | | **Total at drug level** |
| --- | --- | --- | --- | --- | --- | --- | --- | --- | --- |
|  | **CYP2C8** | **CYP2C9** | **CYP2C19** | **CYP2D6** | **CYP3A** | **P-gp** | **SLCO1B1** | **VKORC1** |  |
| **i) Number of substantial interactions** | | | | | | | | | |
| **Substrates** | 13 | 98 | 894 | 128 | 651 | 1240 | 614 | 32 | **1840** |
| **Inhibitors** | 552 | 0 | 140 | 15 | 45 | 40 | 1 | - | **654** |
| **Inducers** | 0 | 0 | 0 | - | 7 | 8 | - | - | **8** |
| **Every DDI^a^** | 13 | 0 | 114 | 4 | 45 | 79 | 1 | - | **252** |
| **Simple DDI^b^** | 13 | 0 | 86 | 4 | 33 | 72 | 1 | - | **205** |
| **Every DGI^a^** | - | 2 | 26 | 5 | 0 | - | 15 | 5 | **53** |
| **Simple DGI^b^** | - | 1 | 19 | 4 | 0 | - | 13 | 3 | **40** |
| **DDGI** | - | 1 | 59 | 3 | 0 | - | 28 | - | **91** |
| **DGGI** | - | 4 | 6 | 6 | 0 | - | 0 | 4 | **10** |
| **Interaction total^c^** | **13** | **6** | **170** | **17** | **33** | **72** | **42** | **7** | **346** |
| **ii) Number of patients with at least one substantial interaction per gene** | | | | | | | | | |
| **Every DDI^a^** | 13 | 0 | 108 | 4 | 44 | 45 | 1 | - | **186** |
| **Simple DDI^b^** | 13 | 0 | 82 | 4 | 32 | 44 | 1 | - | **154** |
| **Every DGI^a^** | - | 2 | 25 | 5 | 0 | - | 15 | 5 | **50** |
| **Simple DGI^b^** | - | 1 | 18 | 4 | 0 | - | 13 | 3 | **38** |
| **DDGI** | - | 1 | 59 | 3 | 0 | - | 28 | - | **88** |
| **DGGI** | - | 4 | 6 | 6 | 0 | - | 0 | 4 | **10** |
| **Patient total^d^** | **13** | **6** | **159** | **17** | **44** | **45** | **42** | **7** | **252** |

This table presents, i) the number of substantial interactions, and; ii) the number of patients with at least one substantial interaction mediated per enzyme/transporter, from 652 post-NSTE-ACS patients.

^a^ = ‘Every’ DDI and DGI rows incorporate each identified substantial DDI/DGI, including those that are constituents of substantial DDGIs/DGGIs. Nevertheless, substantial DDIs or DGIs that are a component of an ineligible DDGI (i.e. a DDGI where the constituent DDI and DGI have opposing effects on the victim drug) have been excluded in these rows.

^b^ = ‘Simple’ DDI and DGI rows are the same as ‘every’ DDI/DGI rows except that substantial DDIs/DGIs that are constituents of substantial DDGIs/DGGIs have been excluded; thus, only substantial DDIs or DGIs not considered in more complex interactions are included in these ‘simple’ rows.

^c^ = the interaction total adds the different types of substantial interaction together within a given enzyme/transporter column, and only counts composite interactions (DDGIs/DGGIs) once. The column on the far right-hand side of the table provides the total number of interactions at the ‘drug-level’ (see below).

^d^ = the patient total gives the number of patients with at least one type of substantial interaction per enzyme/transporter, and at the drug-level (final column – see below).

Substantial interactions were classified as follows:

- DDIs due to strong inhibitors/inducers (provided they are not part of an ineligible DDGI – see below).
- DGIs due to interactions between drugs with pharmacogenomic clinical recommendations and homozygous/compound heterozygous actionable diplotypes.
- DDGIs and DGGIs are included only if the constituent interactions acted in the *same* direction (thus, DDGIs were excluded if they led to opposing effects on one gene product or two metabolic pathways relevant to a victim drug).

Autoinhibition/autoinduction was not counted as an interaction. Substantial DDGIs mediated through two different gene products (e.g. a *CYP* and a transporter) were assigned to the gene involved in the component DGI. For atorvastatin (*SLCO1B1*) and clopidogrel (*CYP2C19*), a few DDDGIs were identified, which were included within the relevant DDGI row. Each DGGI was reported in the column of both genes involved (e.g. *CYP2C9* and *VKORC1* for warfarin), but was only counted as one interaction. CYP1A2 was excluded from the table because, whilst nine patients were on a CYP1A2 substrate, no substantial interactions were detected. CYP2B6 was omitted as no patients were on a substrate or inhibitor.

As two drugs can interact with one another through more than one enzyme/transporter, the last column provides total numbers of substantial interactions/number of patients with at least one substantial interaction at the important ‘drug level’ rather than at the level of each enzyme/transporter. - = category not applicable (e.g. no actionable pharmacogenomic variant for the gene and so no DGI assessment performed).
