# Supplementary material for: Multimorbidity, polypharmacy, and drug-drug-gene interactions following a non-ST elevation acute coronary syndrome: analysis of a multicentre observational study
Source: BMC Med. 2020 Nov 25;18:367. doi: 10.1186/s12916-020-01827-z (PMC7687685; doi:10.1186/s12916-020-01827-z)
Supplement: Supplementary file 10 — Additional file 10. Table of associations with patients that have at least one identified drug interaction. [file 12916_2020_1827_MOESM10_ESM.docx]

**Additional file 10. Table of associations with patients that have at least one identified drug interaction**

|  | **Patients with ≥1 drug interaction** | | | |
| --- | --- | --- | --- | --- |
|  | **≥1 any interaction**  **(n=503 patients)** | | **≥1 substantial interaction**  **(n=252 patients)** | |
|  | **OR (95% CI)** | **p-value** | **OR (95% CI)** | **p-value** |
| **Univariate analysis** | | | | |
| Age | 1.01 (0.99-1.02) | 0.34 | 1.00 (0.99-1.02) | 0.55 |
| Sex (F vs M) | 1.16 (0.77-1.75) | 0.47 | 1.12 (0.79-1.58) | 0.52 |
| Cardiovascular multimorbidity† | 1.86 (1.22-2.84) | 3.7x10^-3^ | 2.20 (1.59-3.10) | 3.0x10^-6^ |
| Non-cardiovascular multimorbidity† | 2.34 (1.43-3.82) | 7.2x10^-4^ | 1.97 (1.38-2.82) | 1.9x10^-4^ |
| All multimorbidity | 1.89 (1.31-2.74) | 7.1x10^-4^ | 2.38 (1.70-3.33) | 4.5x10^-7^ |
| Total number of drugs | 1.25 (1.15-1.35) | 3.8x10^-8^ | 1.25 (1.18-1.33) | 2.2x10^-13^ |
| **Multivariable analysis** | | | | |
| Total number of drugs | 1.25 (1.15-1.35) | 3.8x10^-8^ | 1.22 (1.15-1.30) | 9.8x10^-10^ |
| All multimorbidity | - | - | 1.55 (1.07-2.24) | 0.019 |

Univariate and multivariable logistic regression was carried out to identify potential predictors of interactions by comparing patients with at least one interaction of any strength (‘All’), or with at least one predicted substantial interaction (‘Substantial’), versus patients without such drug interactions, from within the interaction cohort (n=652). All types of interaction (drug-drug, drug-gene, drug-drug-gene, and drug-gene-gene) were considered when identifying patients with at least one interaction. Multimorbidity was defined as ≥2 comorbidities.

† = not included in multivariable logistic regression modelling as they are constituents of all multimorbidity.
