# Supplementary material for: Multimorbidity, polypharmacy, and drug-drug-gene interactions following a non-ST elevation acute coronary syndrome: analysis of a multicentre observational study
Source: BMC Med. 2020 Nov 25;18:367. doi: 10.1186/s12916-020-01827-z (PMC7687685; doi:10.1186/s12916-020-01827-z)
Supplement: Supplementary file 11 — Additional file 11. Sensitivity analysis after substitution of dichotomous multimorbidity (≥2 comorbidities) with number of comorbidities. [file 12916_2020_1827_MOESM11_ESM.docx]

**Additional file 11. Sensitivity analysis after substitution of dichotomous multimorbidity (**≥2 **comorbidities) with number of comorbidities**

| **Predictor variable** | **Covariate(s) adjusted for** | **Outcome variable** | **Cohort** | **Result** | **P-value** |
| --- | --- | --- | --- | --- | --- |
| **Analyses of numbers of comorbidities and drug use:** | | | | | |
| Age (mean centred) | Sex, Age (mean centred) squared | Number of comorbidities | All | RR 1.02 (1.02-1.03) | <2.0x10^-16^ |
| Sex (F vs M) | Age (mean centred),  Age squared (mean centred) | Number of comorbidities | All | RR 1.02 (95% CI 0.94-1.12) | 0.60 |
| Number of comorbidities | Age | On all five secondary prevention cardiovascular drugs | All | OR 0.81 (95% CI 0.75-0.88) | 2.1x10^-7^ |
| Number of comorbidities | - | Number of drugs | All drugs | RR 1.11 (95% CI 1.09-1.12) | <2.0x10^-16^ |
| Number of comorbidities | Age | Number of drugs | All drugs | RR 1.10 (95% CI 1.08-1.12) | <2.0x10^-16^ |
| **Analyses of patients that have at least one identified interaction:** | | | | | |
| Number of comorbidities | - | ≥1 any interaction | Interaction | OR 1.28 (95% CI 1.12-1.45) | 1.9x10^-4^ |
| Number of comorbidities | Total number of drugs | ≥1 any interaction | Interaction | OR 1.09 (95% CI 0.94-1.26) | 0.26 |
| Number of comorbidities | - | ≥1 substantial interaction | Interaction | OR 1.33 (95% CI 1.20-1.48) | 1.1x10^-7^ |
| Number of comorbidities | Total number of drugs | ≥1 substantial interaction | Interaction | OR 1.12 (95% CI 0.99-1.26) | 0.071 † |
| **Analyses of time to MACE and time to ACM:** | | | | | |
| **Univariate:** | | | | | |
| Number of comorbidities | - | MACE | Interaction | HR 1.39 (95% CI 1.24-1.55) | 5.8x10^-9^ |
| **Multivariable:** | | | | | |
| Age | Number of comorbidities, Total number of drugs | MACE | Interaction | HR 1.05 (95% CI 1.03-1.06) | 2.0x10^-6^ |
| Number of comorbidities | Age,  Total number of drugs | MACE | Interaction | HR 1.22 (95% CI 1.07-1.39) | 2.9x10^-3^ |
| Total number of drugs | Age,  Number of comorbidities | MACE | Interaction | HR 1.08 (95% CI 1.01-1.14) | 0.015 |
| **Univariate:** | | | | | |
| Number of comorbidities | - | ACM | Interaction | HR 1.32 (95% CI 1.15-1.52) | 6.8x10^-5^ |
| **Multivariable:** | | | | | |
| Age | Total number of drugs | ACM | Interaction | HR 1.08 (95% CI 1.06-1.11) | 3.7x10^-11^ |
| Total number of drugs | Age | ACM | Interaction | HR 1.12 (95% CI 1.05-1.19) | 4.0x10^-4^ |
| Number of comorbidities | Age,  Total number of drugs | ACM | Interaction | HR 1.12 (95% CI 0.95-1.31) | 0.18 |

Cohorts: All = the whole cohort (n=1456); All drugs = cardiovascular and non-cardiovascular drugs both known (n=698); Interaction = the interaction cohort with all drugs and relevant genomic variants known (n=652)

CI = confidence interval; HR = hazard ratio; OR = odds ratio; RR = relative risk

† = When all multimorbidity (≥2 comorbidities) was replaced by number of comorbidities results remained equivalent, although number of comorbidities was borderline statistically significant (adjusted p=0.071) in the multivariable logistic regression that compared patients with ≥1 substantial interaction (vs patients with no substantial interaction), whereas multimorbidity was statistically significant (adjusted p=0.019).
